# RAD54 family translocases counter genotoxic effects of RAD51 in human tumor cells

**DOI:** 10.1093/nar/gkv175

**Published:** 2015-03-12

**Authors:** Jennifer M. Mason, Kritika Dusad, William Douglass Wright, Jennifer Grubb, Brian Budke, Wolf-Dietrich Heyer, Philip P. Connell, Ralph R. Weichselbaum, Douglas K. Bishop

**Affiliations:** 1 Department of Radiation and Cellular Oncology, University of Chicago, Cummings Life Science Center, Box 13, 920 East 58th St., Chicago, IL 60637, USA; 2 Department of Microbiology and Molecular Genetics, University of California, Davis, Davis CA 95616, USA; 3 Department of Molecular and Cellular Biology, University of California, Davis, Davis CA 95616, USA; 4 Department of Molecular Genetics and Cell Biology, University of Chicago, Chicago, IL 60637, USA

## Abstract

The RAD54 family DNA translocases have several biochemical activities. One activity, demonstrated previously for the budding yeast translocases, is ATPase-dependent disruption of RAD51-dsDNA binding. This activity is thought to promote dissociation of RAD51 from heteroduplex DNA following strand exchange during homologous recombination. In addition, previous experiments in budding yeast have shown that the same activity of Rad54 removes Rad51 from undamaged sites on chromosomes; mutants lacking Rad54 accumulate nonrepair-associated complexes that can block growth and lead to chromosome loss. Here, we show that human RAD54 also promotes the dissociation of RAD51 from dsDNA and not ssDNA. We also show that translocase depletion in tumor cell lines leads to the accumulation of RAD51 on chromosomes, forming complexes that are not associated with markers of DNA damage. We further show that combined depletion of RAD54L and RAD54B and/or artificial induction of RAD51 overexpression blocks replication and promotes chromosome segregation defects. These results support a model in which RAD54L and RAD54B counteract genome-destabilizing effects of direct binding of RAD51 to dsDNA in human tumor cells. Thus, in addition to having genome-stabilizing DNA repair activity, human RAD51 has genome-destabilizing activity when expressed at high levels, as is the case in many human tumors.

## INTRODUCTION

The strand exchange protein RAD51 functions to promote genome stability by repairing DNA double strand breaks (DSB) and damaged replication forks (1–3). RAD51 repairs damage by forming helical nucleoprotein filaments on tracts of ssDNA. Such tracts form by 5′-3′ processing of DNA ends formed by DSBs, and also as a consequence of replication fork collapse or blockage. The ssDNA-specific binding protein RPA binds rapidly and with high specificity to ssDNA tracts and, with the help of mediator proteins, promotes the recruitment of RAD51 (reviewed by (4)). Following nucleoprotein filament formation, RAD51 carries out a search for homologous dsDNA sequences and then promotes invasion of target duplex leading to the exchange of DNA strands that forms heteroduplex DNA within an intermediate called the displacement loop (D-loop). The ssDNA strand displaced from the target duplex during heteroduplex DNA formation also binds RPA (5). Subsequent stages of the recombination process result in repair of damage without loss or rearrangement of DNA sequences. RAD51 complexes engaged in repair can be detected by immunostaining and light microscopy and are visualized most often as foci, i.e. structures smaller than the resolution limit of light microscopy. RAD51 focus formation can be induced by treatments that damage DNA or inhibit replication, and the majority of these damage-induced RAD51 foci co-localize with RPA.

Despite this central role in homology-mediated repair and genome stabilization, high levels of RAD51 expression can result in reduced proliferation and increased genomic instability (6,7). Intriguingly, RAD51 is commonly expressed at relatively high levels in human tumor cells compared to noncancerous cells and the nuclei of these cells contain elevated levels of spontaneous RAD51 foci compared with nontumor cells (8–14). Increased spontaneous RAD51 nuclear foci were observed in cell lines derived from a wide variety of cancers including acute myeloid leukemia, T-cell lymphoma, breast carcinoma and melanoma.

The RAD54 family of DNA translocase proteins function in concert with RAD51 to promote recombinational DNA repair (reviewed by (15)). These proteins are members of the Swi2/Snf2 family of motor proteins that utilize energy from ATP hydrolysis to translocate on dsDNA, but not ssDNA (16–20). Dissociation of RAD51 from dsDNA is thought to be important to clear the 3′ ends of invading ssDNAs of RAD51 during recombinational repair, thereby allowing DNA polymerases to use 3′ ends as primers for the DNA repair synthesis required to complete the repair process (21). RAD54 translocation has also been proposed to act following homology recognition as a ‘heteroduplex pump’ to incorporate the invading ssDNA into the D-loop while simultaneously removing RAD51 during the generation of the heteroduplex product (22). D-loop formation is associated with local chromatin remodeling *in vivo* (23–27) and biochemical data shows that RAD54 translocation displaces nucleosomes (28). Not only has RAD54 been shown to remove RAD51 from dsDNA, it has also been reported to stabilize the interaction of RAD51 with ssDNA *in vitro* by a process that does not require ATP hydrolysis (29). This activity can be observed by anti-RAD51 chromatin immunoprecipitation *in vivo* (30). Thus, RAD54 appears to contribute to DNA repair by stabilizing association of RAD51 with ssDNA prior to RAD51-mediated strand exchange and then disassembling RAD51 from the dsDNA exchange product.

In addition to pro-recombinogenic activities of Rad54 family translocases, studies in budding yeast have shown that the translocases prevent accumulation of nonrepair-associated DNA bound forms of Rad51 and its meiosis-specific paralog Dmc1 (31,32). In the absence of translocase activity, Rad51 accumulates on undamaged chromosomes causing growth arrest and chromosome loss (31). The activity of Rad54 family translocase activity in removing Rad51 from undamaged dsDNA provides an interesting contrast to the activities of other helicase/translocase proteins such as FANCJ, FBH1, BLM or RECQ5, which remove RAD51 from ssDNA. This mechanism prevents homologous recombination in situations where such recombination is unnecessary and potentially detrimental (reviewed by (33)).

Our studies on translocase function in budding yeast raised the possibility that mammalian translocases, RAD54L and RAD54B, could also function to prevent the accumulation of nondamage-associated RAD51 complexes. In mice, RAD54L and RAD54B deficiency or expression of a dominant-negative RAD54 allele display elevated levels of RAD51 foci on DNA (34,35). This elevation in spontaneous RAD51 foci was interpreted as resulting from defective repair at sites of DNA damage. However, our results from budding yeast led us to hypothesize that at least some of these spontaneous RAD51 foci might represent nondamage-associated complexes resulting from an imbalance between RAD51 and RAD54 translocase activity.

The accumulation of RAD51 on undamaged DNA has important implications for cancer etiology. RAD51 steady-state levels are elevated in a wide variety of tumor cells. The tumor cells may be susceptible to toxic accumulation of RAD51, if high levels of RAD51 result in an imbalance of RAD51 and RAD54 activity. We previously showed that a compound called RS-1, which enhances binding of RAD51 to DNA, causes accumulation of nondamage-associated RAD51 complexes and cell death (36). We also showed that depletion of RAD54 translocases sensitized cells to RS-1 treatment. However, it was not determined if human translocases function to suppress RAD51 accumulation on undamaged DNA.

Here we demonstrate that RAD54 translocase depletion and/or RAD51 overexpression in human tumor cells results in the accumulation of RAD51 foci that do not co-localize with markers of DNA damage. Accumulation of these foci is associated with interruptions in replication, defective chromosome segregation and reduced proliferation. We also show that RAD54 protein dissociates RAD51 from dsDNA, but not ssDNA, using purified protein, as shown previously for the yeast ortholog. Given that many human tumor cells express unusually high levels of RAD51, the results have potential implications with respect to both the mechanism of tumor progression and cancer therapy.

## MATERIALS AND METHODS

siRNA transfection conditions, determination of RAD51 cellular concentration, RAD51 DNA-binding assay, RAD51 displacement assay and antibodies used in this study are described in Supplementary Materials.

### Cell culture

HT1080 (S33) cells were cultured in DMEM (Invitrogen) supplemented with 10% fetal bovine serum, 1 mM sodium pyruvate, 4× nonessential amino acids, 1× penicillin/streptomycin, 100 μg/ml G418, 50 μg/ml hygromycin B and 5 ng/ml doxycycline. MCF7 and MDAMB231 cells were cultured in DMEM containing 10% fetal bovine serum and 1× penicillin/streptomycin. PC-3 cells were cultured in DMEM/F12 supplemented with 10% fetal bovine serum. Cells were cultured at 37°C with 5% CO_2_. Wild-type and *Rad54l*-/-*Rad54b* -/- mouse embryonic stem cells (35) were cultured on gelatin-coated dishes in GMEM media (Sigma) supplemented with 15% EmbryoMax FBS (Clontech), 4× nonessential amino acids, 1× penicillin/streptomycin, 1 mM sodium pyruvate, 2 mM L-glutamine, 50 mM 2-mercaptoethanol and 1000 U/ml leukemia inhibitory factor (LIF).

### Western analysis

Western analysis of RAD51 was done as previously described (36).

### Immunofluorescence

Detection of proteins by immunofluorescence was done as previously described (36) with the following changes. Cytosolic proteins were pre-extracted with HEPES/Triton X-100 buffer (20 mM HEPES pH 7.4, 0.5% Tritron X-100, 50 mM NaCl, 3 mM MgCl_2_, 300 mM sucrose) prior to fixation in 3% para-formaldehyde, 3.4% sucrose in ddH_2_O, pH 7.0 for 10 min at room temperature. For the DNase experiments, cells were incubated with 15 μg/ml DNaseI in 0.2% Triton X-100, 200 mM PIPES pH 6.8, 1 mM MgCl_2_ for 60 min at room temperature prior to fixation. Cells were irradiated with 6 Gy ionizing radiation 6 h prior to fixation using a Maxitron generator. To image cells, 0.5–1 micron sections of the cells were acquired on a scanning laser microscope (LSM510, Zeiss) using LSM software with a 60× objective. Previous studies examining spontaneous RAD51 foci observed cells containing >10 discrete foci as well as cells with fewer, but larger foci (14). To encompass both classes of RAD51 foci, cells containing ≥ five discrete foci were considered focus positive. Brightness and contrast were adjusted using ImageJ. Total cellular fluorescence = integrated density - (area of interest X mean background level) (37). For RAD51 fiber quantitation, RAD51 fibers were counted in z slices separated by one micron to minimize the number long fibers that transverse more than one slice. The number of RAD51 fibers is the sum of fibers from z slices. In S33 cells, RAD51 and RPA foci were quantitated from 50 nuclei from two independent experiments. In MCF7 cells, RAD51 and RPA foci were quantitated from 100 nuclei from two independent experiments. For experiments examining mitotic defects, 50 nuclei from two independent experiments were examined in both MCF7 and S33 cells.

### Nuclear abnormalities

Nuclei stained with DAPI were examined for nuclear aberrations. Cells were examined for the presence of micronuclei and DNA bridges. Multinucleated cells were cells containing more than one large nuclear staining body as well as cells with a multilobed staining body. In S33, 50 random nuclei from two independent experiments were examined for nuclear anomalies. To determine if the more severe nuclear aberrations were present in a low percentage of MCF7 cells, 300 nuclei from three independent experiments were examined.

### EdU growth assay

At the indicated times post-transfection, EdU was added to the culture media to a final concentration of 10 μM and cells were stained for EdU as per manufacturer's instructions (Invitrogen, Click-IT). Images were acquired using a Zeiss Axiovision epifluorescence microscope at 100X magnification. EdU staining was quantitated from 150 nuclei from three independent experiments in S33 and MCF7 cells.

### Nascent DNA assay

DNA fibers were prepared and immunostained as previously described (38). Images were acquired using a Zeiss Axiovision epifluorescence microscope at 100X magnification. The lengths of IdU (red) and CldU (green) tracts were measured using ImageJ software. At least 250 fibers were measured for each experimental condition.

### Statistical analyses

For focus counts and CldU/IdU replication tract experiments, *P*-values were calculated using Wilcoxon Rank Sum Test. For EdU incorporation and chromosome segregation, *P*-values were calculated using the F-test.

## RESULTS

### RAD51 overexpression results in the accumulation of DNA-associated RAD51 complexes

**Figure 1.**
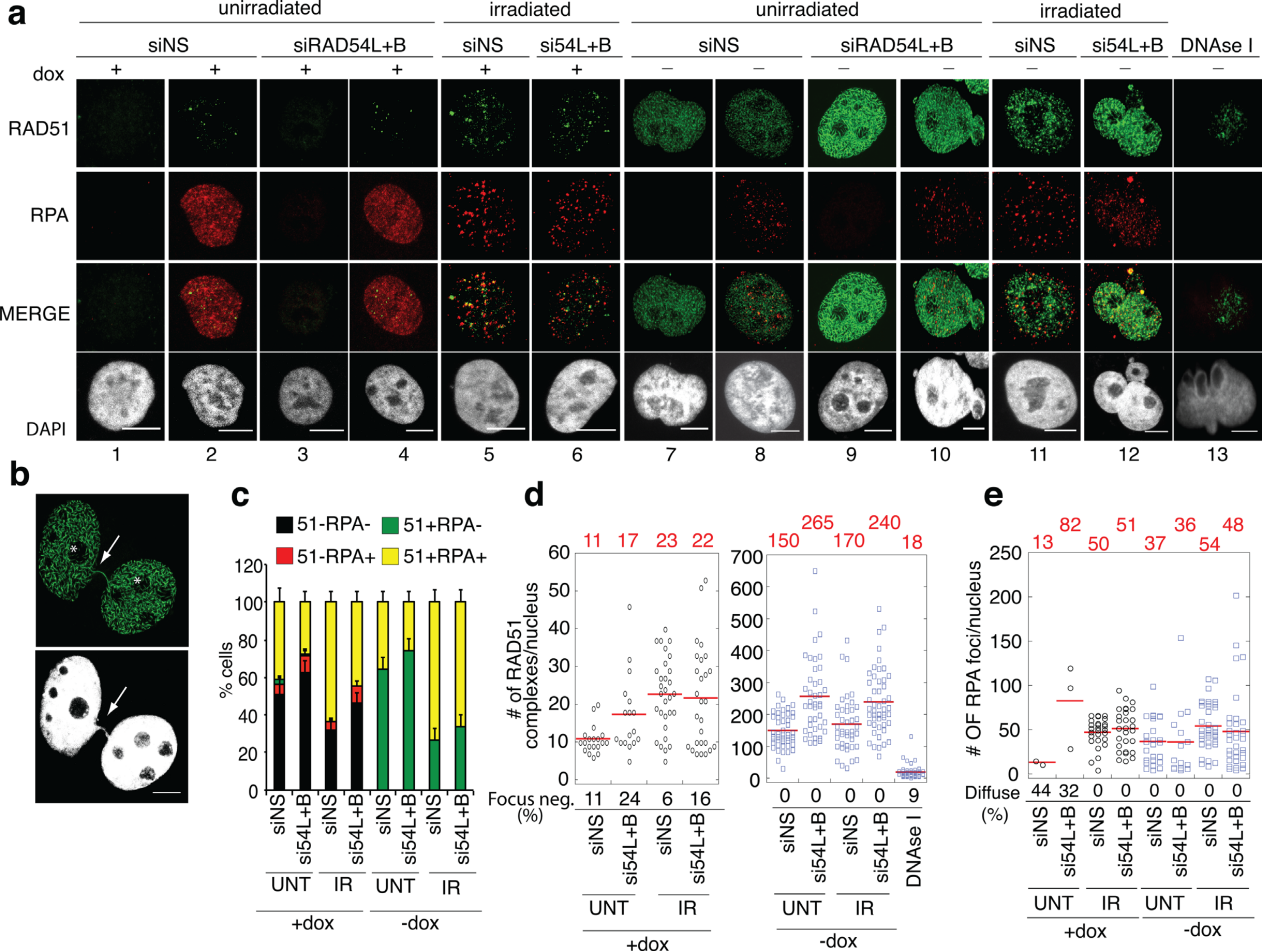
RAD54 translocase depletion increases RAD51 complex formation in S33 cells overexpressing RAD51. **(a)** Representative images of S33 cells after the indicated treatments. RAD51 (green), RPA (Red), DNA (blue). RAD51 staining in RPA-negative cells (columns 1 and 3) and RPA-positive cells (unirradiated columns 2 and 4, irradiated columns 5 and 6). RAD51 staining in RPA-negative cells (columns 7 and 9) and RPA-positive cells (unirradiated columns 8 and 10, irradiated columns 11 and 12) or after treatment with DNase I (column 13) in cells overexpressing RAD51. **(b)** Representative images of a nucleus containing extensive RAD51 fibers. A DAPI staining thread that connects two nuclei and co-localizes with a RAD51 fiber is marked with an arrow and nucleoli are indicated with asterisks. **(c)** Graph depicts fraction of cells that were RAD51 and/or RPA focus positive. Error bars, S.E. **(d)** Dot plots depicting the number of RAD51 foci in RAD51 focus-positive cells/RPA-positive cells cultured in the presence of dox (left) or the number RAD51 fibers in cells cultured in the absence of dox (right). Red line and numbers above the graph represents mean focus counts. The percentage of the population that was RAD51 focus-positive is depicted below the graph. **(e)** Dot plot depicting number of RPA foci in RPA-focus positive nuclei after the indicated treatments. The percentage of the population containing S-phase RPA staining patterns is indicated below the graph. NS, nonsilencing. Scale bars, 10 μm.

To characterize the nuclear localization of RAD51, we began with the S33 cell line, a derivative of the human fibrosarcoma line HT1080 that carries an untagged RAD51 expression construct under the control of a doxycycline (dox)-repressible promoter (6). Growth of S33 cells in media containing 5 ng/ml dox maintains relatively low RAD51 expression; removal of dox increases RAD51 protein level 60-fold from 0.06 to 4 μM (Supplementary Figure S1a). Previous studies showed that induction of RAD51 in S33 reduces proliferation and results in formation of elongated RAD51 fibers (6). We extended these findings using an immunostaining method that extracts soluble nuclear protein, leaving only insoluble protein, including protein bound to chromosomes (39). Removal of dox (i.e. overexpression of RAD51) resulted in an increase in both the number of RAD51 complexes/nucleus from 11 ± 3 RAD51 foci per focus-positive cell (defined as > five foci) to 150 ± 53 and the lengths of these structures from less than the resolution limit (∼0.2 μm) to 1.2 ± 0.7 μm (Figure 1a). In 8% of nuclei, fiber formation was even more extensive, with neighboring fibers usually intersecting in Z-axis projections (Figure 1b). Interestingly, RAD51 fibers appeared to be excluded from nucleoli, which were visualized as DAPI-negative subnuclear regions (Figure 1b). Importantly, DNase I treatment of RAD51 overexpressing cells reduced the number of RAD51 fibers over 8-fold to 18 ± 21 (Figure 1a,d). DNase treatment also caused a dramatic loss in staining for the replisome clamp protein PCNA, which binds dsDNA. In contrast, DNase treatment did not alter the staining pattern of the nuclear matrix protein Lamin B, a protein that does not associate with DNA (Supplementary Figure S2a) (40,41). These results indicate that RAD51 fibers that form as a consequence of overexpression represent a DNA bound form of the protein. Another observation indicating association of the fibers with DNA was detection of thin DNA-staining threads (bridges) connecting two adjacent nuclei. RAD51 localizes to the contour of these DNA threads, a staining pattern strongly suggesting that fibers are bound to DNA (Figure 1b and Supplementary Figure S2c, see also Figure 6a below).

**Figure 2.**
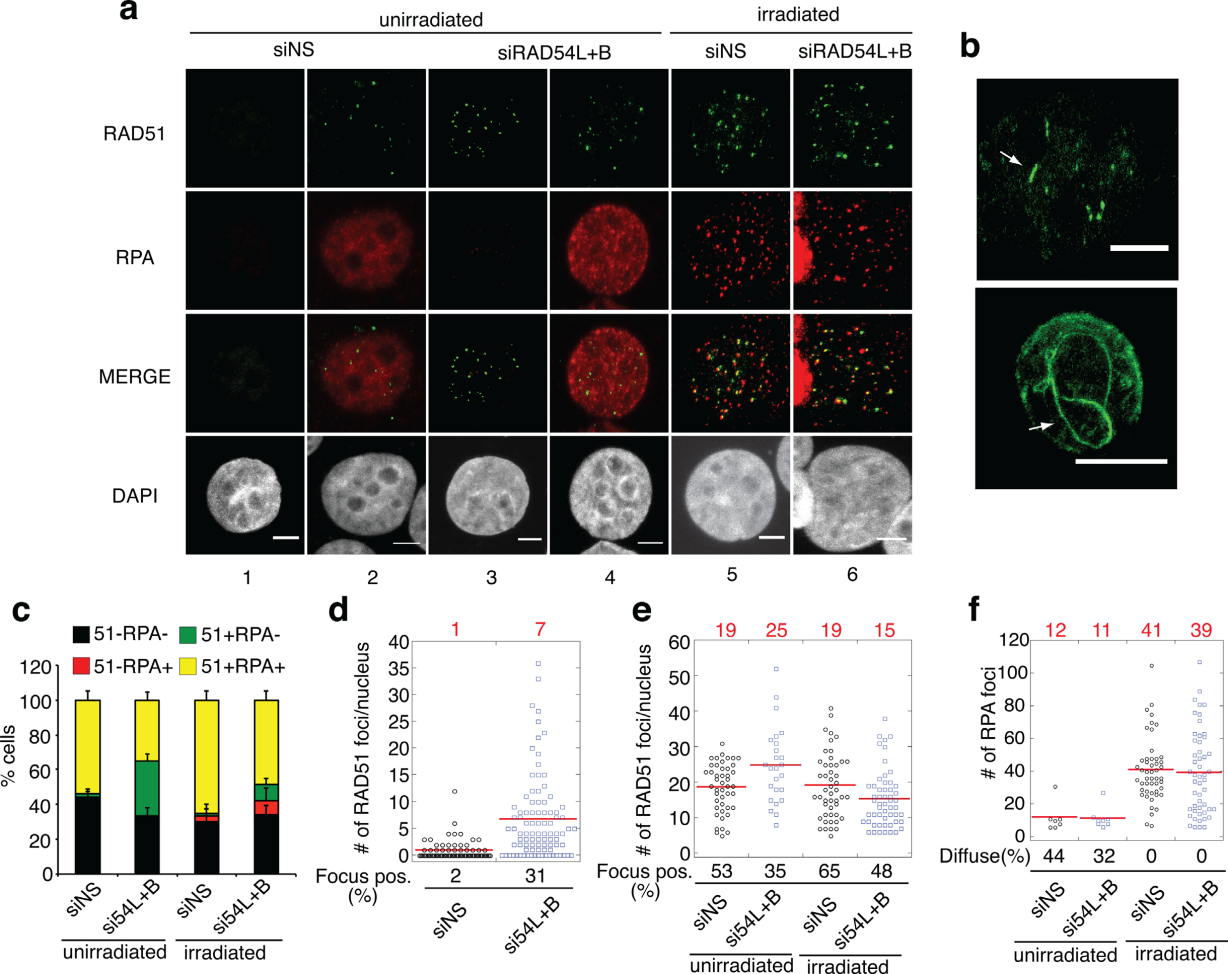
RAD54 translocase depletion increases RAD51 focus formation in MCF7 cells. **(a)** Representative images of nuclei after indicated treatments. Staining was carried out as described in legend to Figure 1. Images depict non-S phase nuclei (columns 1 and 3) and RPA-positive (S-phase) nuclei (unirradiated columns 2 and 4, irradiated columns 5 and 6). **(b)** Representative nuclei containing elongated RAD51 staining structures. **(c)** Graph depicts fraction of the cell population that was RAD51 and/or RPA focus positive. **(d)** Dot plot depicting number of RAD51 foci in MCF7 cells. The plot depicts RAD51 focus counts among the non-S phase (RPA-negative) cells. The percentage of the population that was RAD51 focus-positive/RPA-focus negative is depicted below the graph. Red line and numbers above the graph represent the mean RAD51 focus counts. **(e)** Dot plot depicting RAD51 focus counts in RPA-positive cells in unirradiated cells and irradiated cells as in Figure 1. **(f)** Dot plot depicting number of RPA foci in focus-positive MCF7 cells, as in Figure 1. Scale bars, 5 μm.

**Figure 3.**
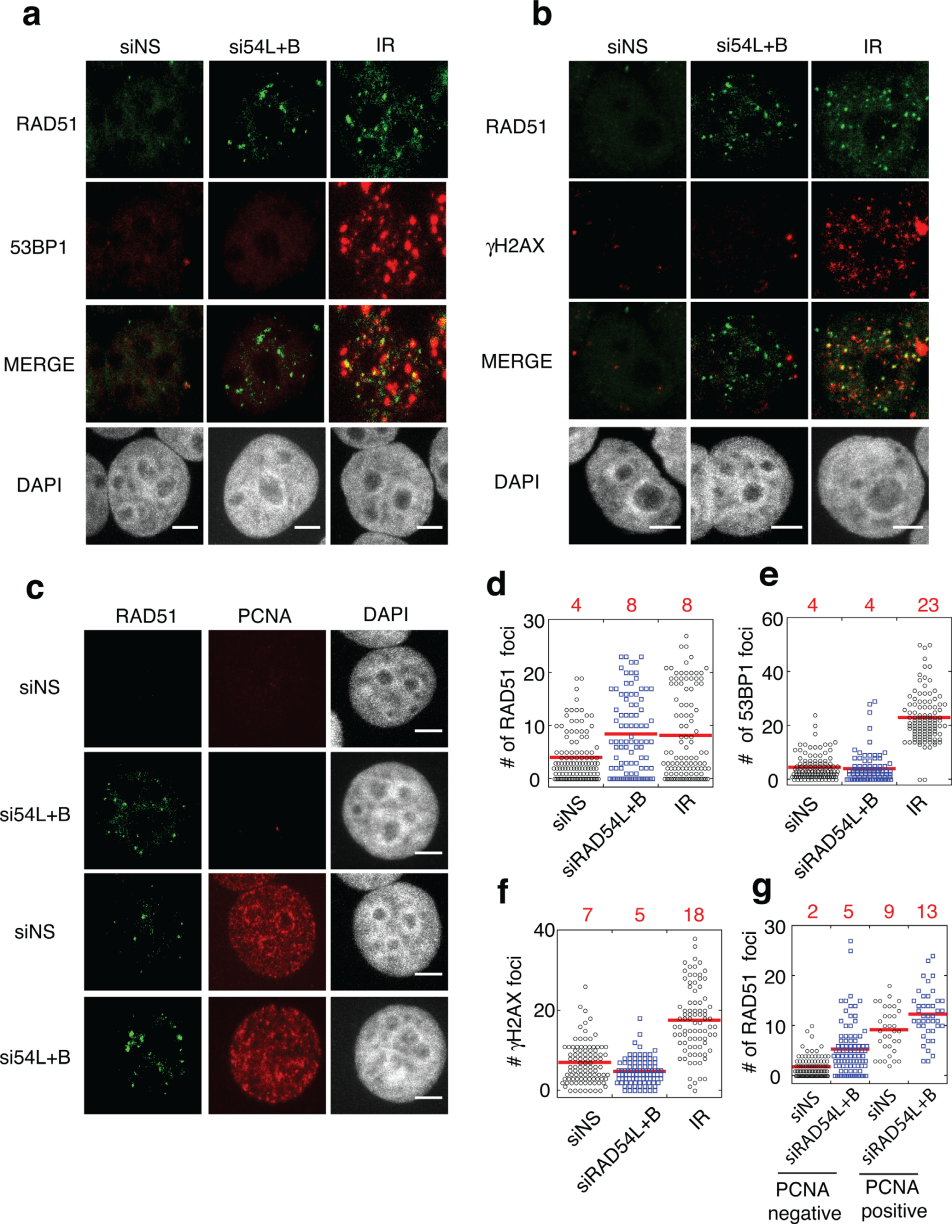
Depletion of translocases does not result in an increase in markers of DNA damage. Representative images of nuclei containing RAD51 foci and either **(a**) 53BP1 foci (**b**) γ-H2AX foci or **(c)** PCNA. (**d-g**) Dot plots representing the number of foci in MCF7 cells after the indicated treatments for **d**) RAD51, (**e**) 53BP1 (**f**) gH2AX (**g**) and RAD51 in PCNA-negative and PCNA-positive nuclei. Red lines and numbers above the graphs represent the mean RAD51 focus counts. Scale bar; 5 μm.

**Figure 4.**
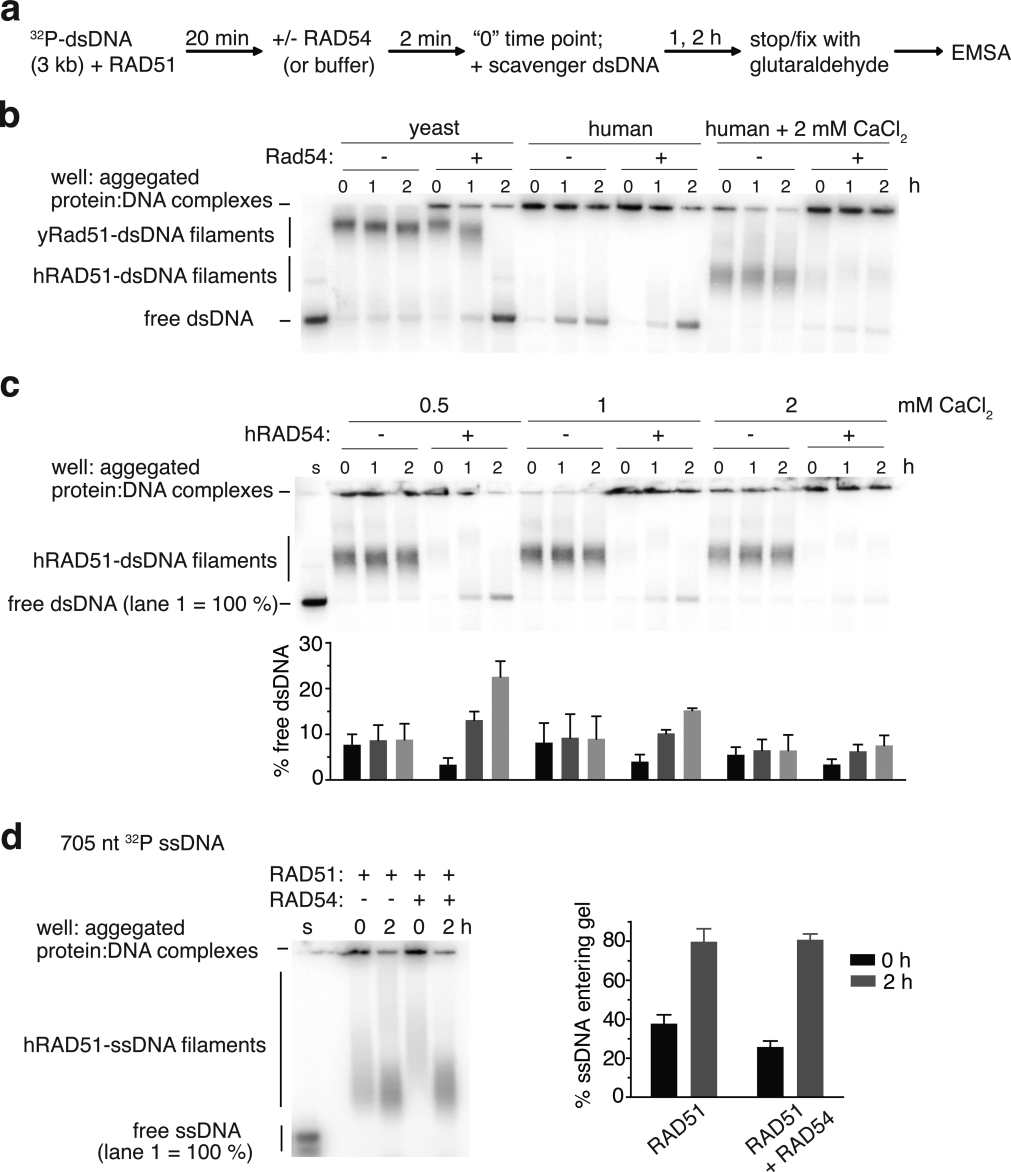
Human RAD54 disassembles human RAD51-dsDNA filaments *in vitro*. **(a)** Scheme of electrophoretic mobility shift assay (EMSA) for RAD54-mediated disassembly of RAD51-dsDNA filaments. A 3 kilobase dsDNA substrate (6 μM bp) that is 5′ end-labeled with ^32^P is incubated with RAD51 (1.5 μM) for 20 min at room temperature (∼23°C). RAD54 (100 nM) is then added, or identical buffer, and incubation continued for 2 min. A 30 μM bp pUC19 ‘scavenger’ dsDNA is then added to bind RAD51 protein that is removed by RAD54, preventing rebinding to the original dsDNA. After 1 or 2 h, the reaction is stopped by fixation with 0.25% glutaraldehyde and subject to TAE-agarose gel electrophoresis. **(b)** Phosphorimage of EMSA performed with human or yeast Rad51+/- Rad54 cognate pairs. Reactions contained 4 mM MgCl_2_ and CaCl_2_ where indicated. **(c)** Calcium titration into the disassembly reaction, with 4 mM magnesium ion and the indicated concentration of calcium. Quantitation corresponding to each lane condition is provided underneath the gel image. The first lane (marked ‘s’) contains DNA substrate and no protein and represents 100% signal to which the other lanes’ free DNA are normalized. Error bars are the standard deviation of three independent experiments. **(d)** Experiment performed with a 705 nt ssDNA substrate (6 μM nucleotide) and 0.5 mM CaCl_2_. The doublet band seen in the substrate only lane, marked ‘s’, is likely due to alternative secondary structural forms of the ssDNA substrate. Error bars are the standard deviation of three independent experiments.

**Figure 5.**
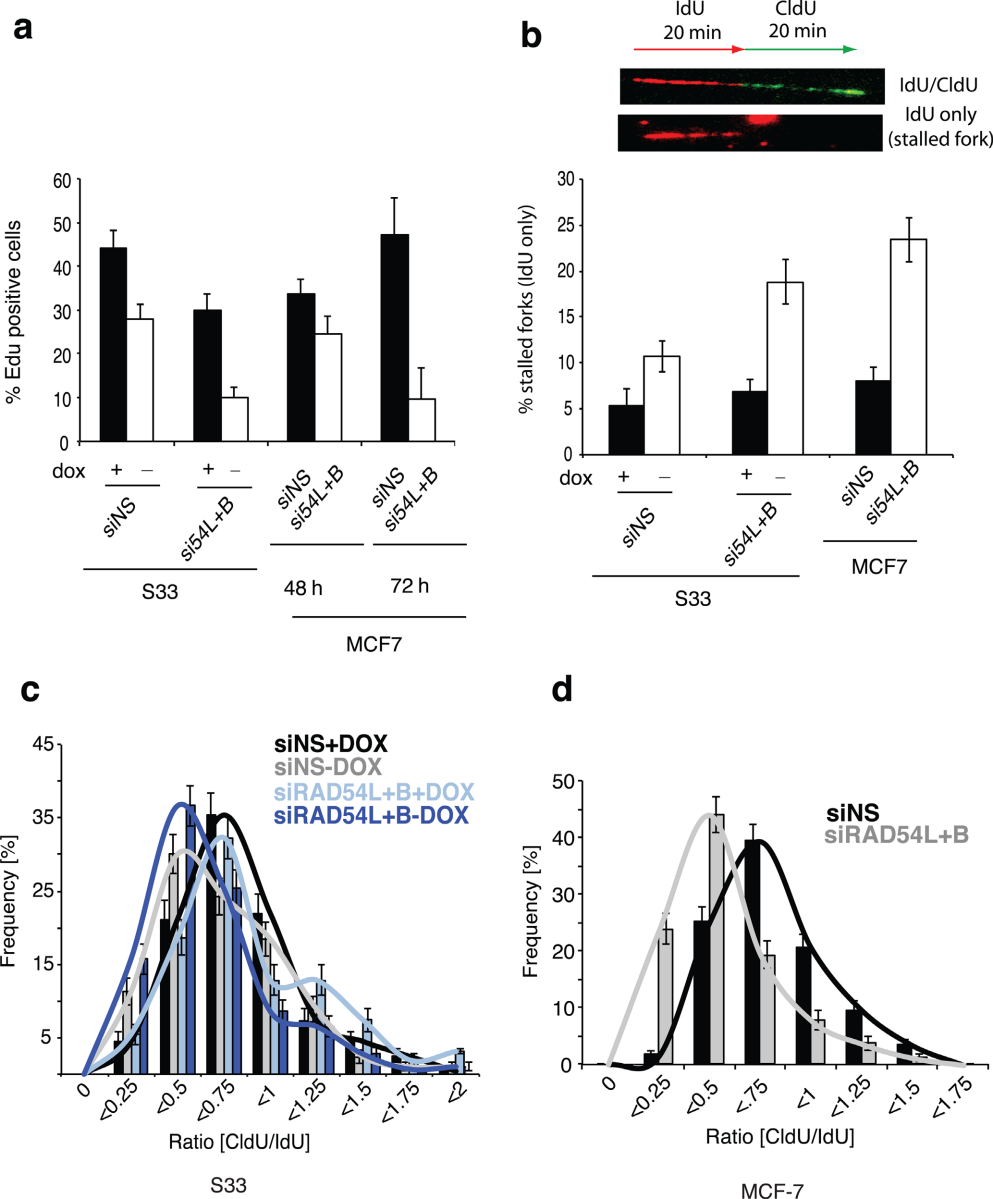
Proliferation and replication defects in S33 and MCF7 cells after RAD54 translocase depletion. (**a**) EdU assay for cell proliferation. Graph represents the percentage of cells that incorporated EdU in S33 cells (48 h) and MCF7 (48 and 72 h) after the indicated treatments. Note that reductions in the percent of EdU-positive cells can result from blocks at any phase of the cell cycle. (**b**) Quantification of stalled replication forks in S33 cells and MCF7 cells using the DNA fiber spreading technique. Images above graph represent a replication tract that has incorporated both IdU (red) and CldU (green) during consecutive pulses and a tract that incorportated IdU only (stalled fork). (**c**) Replication fork progression in (c) S33 cells and (**d**) MCF7 cells. The ratio of CldU/IdU tracts are plotted. Error bars, SE.

**Figure 6.**
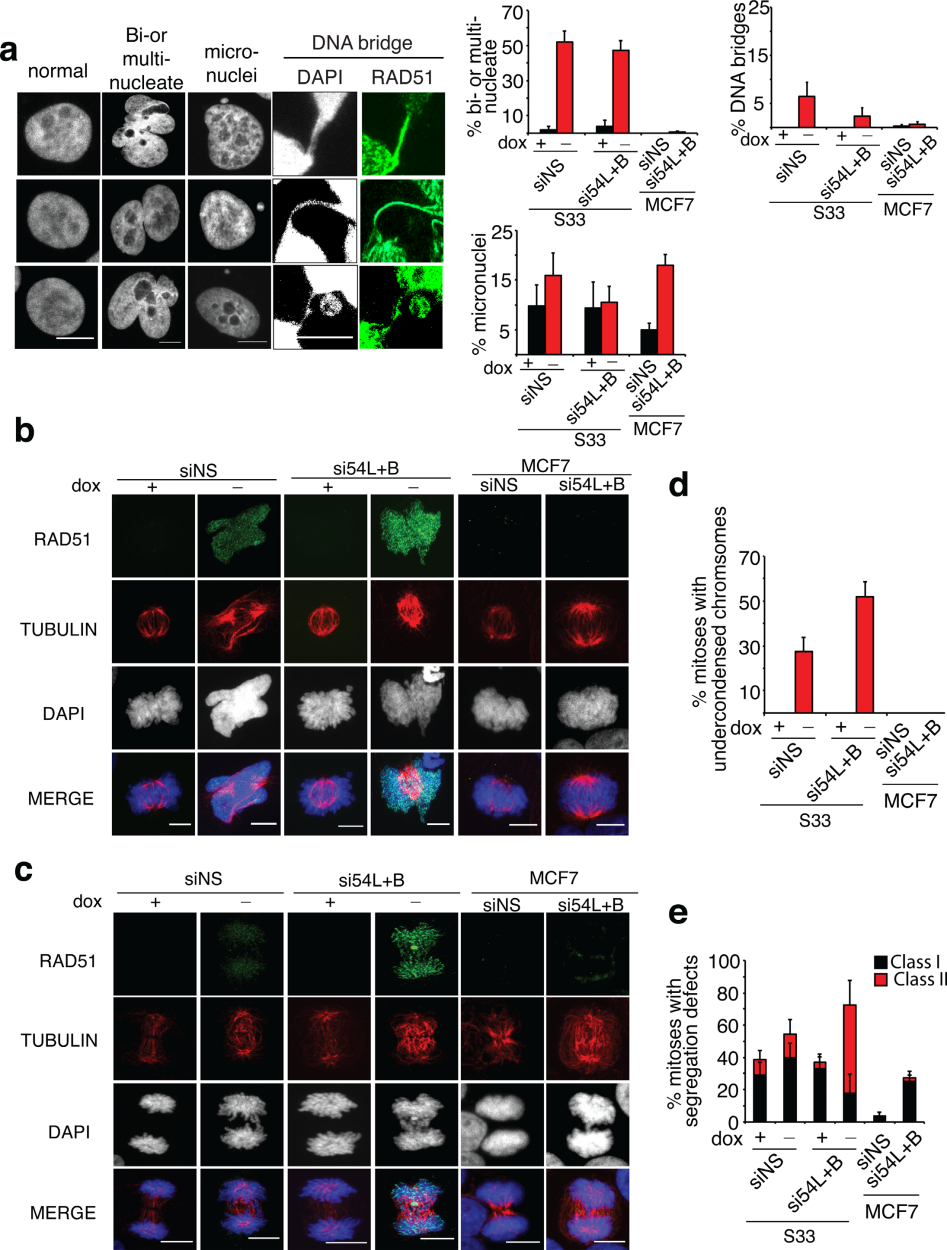
Mitotic defects in S33 and MCF7 cells after RAD54 translocase depletion. **(a)** Representative images of S33 cells and/or MCF7 cells. Three independent images of each type of abnormality are shown. Graphs depict quantification of nuclear aberrations. **(b)** Representative images of metaphases in S33 and MCF7 cells after indicated treatments. Cells were stained for RAD51 (green) and α-Tubulin (red). **(c)** Representative images of anaphases in S33 and MCF7 cells after indicated treatments. **(d)** Quantification of mitotic figures displaying chromosome condensation defects. **(e)** Percentage of mitoses with Class I or Class II segregation defects. Error bars, SE. NS, nonilencing. Scale bar, 10 μm.

DNA bridges can arise as a result of catenated DNA persisting through anaphase. Previous studies have shown that the centromere protein, PICH, localizes to catenated DNA (42). We examined localization of PICH on DNA bridges in RAD51 overexpressing cells. We observed PICH staining DNA bridges in approximately 52 ± 9% of nuclei (Supplementary Figure S2b). In 60% of PICH-positive DNA bridges, PICH was localized only on a fraction of the DNA bridge. No DNA bridges between interphase nuclei were observed in cells cultured in the presence of dox (data not shown). Thus, at least a subset of DNA bridges are likely to arise due to the failure to resolve catenated DNA.

Previous studies did not determine whether or not RAD51 fibers induced by RAD51 overexpression are associated with tracts of ssDNA, as would be expected if the fibers were assembled at sites of damage. To address this, S33 cells were co-stained with antibodies against RAD51 and the ssDNA binding protein, RPA. In unperturbed S phase cells, RPA displays a characteristic staining pattern comprising domains of relatively bright staining on a background of diffuse staining of intermediate intensity; RPA staining is minimal or absent in G2, M and G1-phases (43–45). Following induction of replication stress or DNA breaks, RPA is redistributed within S-phase cells forming a punctate pattern (46,47).

We performed RPA-RAD51 double staining in S33 cells with or without induction of RAD51 overexpression. RAD51 overexpression induced a similar level of RAD51 foci in cells with extensive RPA staining (150 ± 56 RAD51 foci/nucleus) and in cells that contained little or no RPA staining (151 ± 52 RAD51 foci/nucleus). (NB: RPA staining-negative cells were defined as those with less than five RPA staining foci with no diffuse background pattern.) The RPA staining-positive cells displayed the punctate pattern typically observed after replication stress or DNA damage (Figure 1a,c,e) rather than the more diffuse S-phase pattern observed in control cells. Only 35 ± 6% of RAD51 fiber-containing nuclei were RPA staining-positive. Thus, in more than half of the cells, extensive RAD51 foci and fibers accumulate without associated RPA foci. These data strongly suggest that overexpression of RAD51 can cause accumulation of RAD51 foci at sites that do not contain ssDNA. Within the RPA-positive subpopulation, induction of RAD51 overexpression was associated with redistribution of RPA from the diffuse staining pattern to punctate foci, suggesting that either RAD51 fibers initiate directly on ssDNA at replication forks or that they form on undamaged dsDNA, with subsequent local accumulation of RPA foci as a consequence of replisome collisions with preformed RAD51 fibers. We favor the latter possibility because, as mentioned above, RAD51 fibers were present in similar numbers in both RPA-positive and RPA-negative cells. It should also be noted that treatments which block DNA polymerase activity cause a similar conversion of RPA staining patterns (46,48,49).

### Translocase depletion enhances accumulation of RAD51 complexes in S33 cells overexpressing RAD51

Previous studies in budding yeast showed that RAD54 translocases function to limit the accumulation of nondamage-associated RAD51 foci (31). To determine if RAD54 translocase activity limits RAD51 fiber accumulation in S33 cells, we depleted RAD54L and RAD54B, by 5- and 10-fold respectively, using RNA interference (Supplementary Figure S1 b–g). We examined RAD51 and RPA localization in RAD54L+RAD54B translocase-depleted cells, with or without concurrent overexpression of RAD51. Control cells were transfected with nonsilencing (NS) siRNAs. At lower levels of RAD51 expression, S-phase cells showed a modest 1.5-fold increase in RAD51 foci that was significant when analyzed by the Wilcoxon Rank Sum Test (17 ± 10 versus 11 ± 3, respectively; Figure 1a,d; *P* < 0.005). Thus, RAD54L+RAD54B may limit the number of spontaneous RAD51 foci during S-phase in S33 cells, even without forced overexpression of RAD51. No significant change was detected in non-S-phase cells.

In S33 cells induced to overexpress RAD51, RAD54L+RAD54B depletion increased RAD51 fiber formation containing on average 265 ± 121 RAD51 fibers compared to 150 ± 53 in controls (Figure 1a,d; *P* < 0.001). The fluorescence intensity of DNA-bound RAD51 provided an alternative method to measure the extent of RAD51 complexes. RAD54L+RAD54B depletion resulted in a 2.6-fold increase in RAD51 staining intensity when compared to controls (*P* < 0.005; Supplementary Figure S3a). We verified this result by depleting RAD54L+RAD54B with a second, independent set of siRNAs (Supplementary Figure S1c, siRAD54L+B-2). RAD54L+B-2 depleted cells contained 222 ± 75 RAD51 fibers on average compared to 109 ± 57 fibers in controls (Supplementary Figure S3d; *P* < 0.001). These results indicate that the extent of accumulation of RAD51 fibers following RAD51 overexpression is limited by RAD54 translocases. RAD54L+RAD54B depletion had no impact on steady-state RAD51 levels (Supplementary Figure S1a) indicating that the associated increase in RAD51 fibers resulted from a redistribution of RAD51 in the nucleus.

Next, we determined if these changes in RAD51 focus patterns require depletion of both translocases. Transfection with siRAD54L or siRAD54B did not increase the number of RAD51 fibers; counts were 154 ± 88 and 142 ± 44 foci/nucleus for siRAD54L or siRAD54B, respectively, compared to 153 ± 86 in controls (Supplementary Figure S3b). However, RAD51 fiber length increased from 1.0 ± 0.7 μm in controls to 1.6 ± 0.7 μm and 1.8 ± 0.6 μm in siRAD54L and siRAD54B transfected cells, respectively *(P* < 0.01 and *P* < 0.001*)*. These results indicate that the two translocases share a function that limits the accumulation of RAD51 on DNA in S33 cells expressing high levels of RAD51. The increase in fiber length, but not number, caused by single translocase depletion may indicate that the residual translocase activity in this situation is sufficient to dissociate RAD51–DNA complexes as they initiate, but not sufficient to fully limit fiber elongation.

The results of RPA-RAD51 double staining experiments suggest that RAD51 fibers formed following RAD51 overexpression and/or translocase inhibition are not associated with RPA. To show that very high levels of RAD51 expression do not prevent RPA from forming foci on tracts of ssDNA, we induced such tracts with ionizing radiation and monitored focus induction. Importantly, irradiation increased the fraction of nuclei displaying focal RPA staining patterns 1.6- to 2.6-fold, regardless of RAD51 or RAD54L+RAD54B levels (Figure 1c,e). In cells with lower levels of RAD51, 65–80% of RAD51 foci co-localized with RPA. Although the density of RAD51 fibers precluded accurate estimates of the frequency of RAD51/RPA co-localization, the fraction of RAD51-filament containing nuclei that also contain RPA foci was 2-fold higher than that seen for corresponding conditions without irradiation (Figure 1c,e). This indicates that many unirradiated cells that display RAD51 fibers, but not RPA foci, have the ability to form RPA foci upon induction of DNA damage. Thus, the induction of multiple RAD51 fibers in RPA focus-negative cells by translocase depletion implies that these structures form at sites lacking ssDNA tracts.

### RAD54 family translocases prevent accumulation of nondamage-associated RAD51 complexes in MCF7 cells

RAD51 levels are elevated in a wide variety of human tumor cell lines when compared to nontransformed primary cell lines (8–14). Intriguingly, many tumor lines with elevated RAD51 display spontaneous RAD51 foci (14). Given our results using artificial induction of high levels of RAD51 in S33 cells, we reasoned that spontaneous RAD51 foci observed in other tumor lines might involve binding of the protein to undamaged DNA. We predicted that these cell lines harbor a propensity for RAD51 accumulation that exceeds the cell's ability to remove aberrant RAD51 complexes, i.e. that translocase activity is limiting in these cell lines. If so, reduction of translocase activity would increase the frequency of these RAD51 foci, and generate associated toxicity. We tested this hypothesis in the mammary carcinoma cell line MCF7, which displays relatively high levels of spontaneous RAD51 foci (14). As expected, translocase depletion in MCF7 resulted in a striking increase in RAD51 foci without increasing RPA staining (Figure 2). In RPA-negative cells, the fraction of RAD51 focus-positive cells was 15-fold higher in translocase-depleted compared to control cells (31 ± 5% versus 2 ± 2%, *P* < 0.0005) with an average of 7 ± 3 RAD51 foci/nucleus compared to 1 ± 2 RAD51 foci in controls (Figure 2c,d). A small fraction of the RAD51 structures that accumulated following translocase depletion appeared as fibers (Figure 2b). RAD51 focus counts were also increased in S-phase cells (25 ± 10 in translocase-depleted cells versus 19 ± 7 in control cells; *P* < 0.05; Figure 2e). No change in RPA staining pattern was observed in MCF7 cells following translocase depletion; RPA-staining positive cells displayed the relatively diffuse staining pattern characteristic of unperturbed S-phase rather than the focal staining pattern seen in S33 overexpressing RAD51. Together, these data indicate RAD54 family translocases prevent accumulation of RPA-negative RAD51 foci, especially in non-S-phase cells.

As for S33 cells overexpressing RAD51, MCF7 cells could be induced to form RPA foci by irradiation, even after translocase depletion (Figure 2). We observed a 1.6-fold increase in the RPA staining-positive cell fraction with an average of 39 ± 19 RPA foci/nucleus (Figure 2a,f). Approximately 68–86% of irradiation-induced RAD51 foci co-localized with RPA in both translocase-depleted and control cells. Thus, the RAD51 foci induced by depletion of RAD54L+RAD54B are different from those induced by irradiation in that they do not co-localize with RPA. Again, these results suggest that translocase depletion causes accumulation of RAD51 at sites that do not contain ssDNA.

We observed similar results in *Rad54l*^−/−^*Rad54b*^−/−^ mouse embryonic stem cells (Supplementary Figure S4). These cells were previously reported to have higher than normal levels of RAD51 foci in meiosis, and we found the same to be true during somatic growth (35). As in the human tumor cell experiments, only a small fraction of the RAD51 foci seen in *Rad54l*^−/−^*Rad54b*^−/−^ cells were associated with an RPA focus. Upon irradiation, the majority of RAD51 foci co-localized with RPA. These findings support the conclusion from siRNA experiments that loss of RAD54L+RAD54B function leads to elevated levels of RAD51 foci that are not associated with RPA.

To further support the conclusion that RAD51 foci observed in RAD54L+RAD54B depleted cells are not associated with DNA damage, we examined localization of RAD51 and two key DNA damage markers, 53BP1 and γ-H2AX. Both γ-H2AX and 53BP1 accumulate at DNA ends created by DSBs and collapsed replication forks (50–53). Translocase depletion resulted in a 2-fold increase in the average number of RAD51 foci/nucleus (4 ± 5 NS versus 8 ± 7 siRAD54L+RAD54B; *P* < 0.001; Figure 3a, d). However, RAD54L+RAD54B depletion did not increase the number of 53BP1 (4 ± 5 versus 4 ± 6 foci/nucleus) or γH2AX foci (5 ± 3 versus 7 ± 5 foci/nucleus) compared to controls (Figure 3 a,b,e,f). Furthermore, in RAD54L+B depleted cells, on average only 24 and 23% of RAD51 foci co-localized with 53BP1 and γH2AX, respectively. There is no significant difference in the number of 53BP1 and γH2AX foci between controls and translocase-depleted cells, so the co-localizing foci observed are unlikely to be as a result of increased spontaneous DNA damage. Both γH2AX and 53BP1 foci arise during S phase in unperturbed cells (54,55). Thus, the RAD51 foci in translocase-depleted cells that co-localize with DNA damage markers likely represent stalled/collapsed replication forks. In contrast, irradiation resulted in 3- and 5-fold increases in γH2AX and 53BP1 foci, respectively, and 83–86% of RAD51 foci exhibited co-localization with 53BP1 and γH2AX. This indicates that the majority of RAD51 foci that accumulate following translocase depletion in the absence of irradiation mark undamaged sites.

RAD51 focus formation at DNA damage sites is restricted to S and G2 phases of the cell cycle (56,57). Thus, we determined if RAD51 accumulation in RAD54L+RAD54B depleted cells was occurring in S phase by staining cells with proliferating cell nuclear antigen (PCNA), which stains cells specifically undergoing replication (Figure 3c,g; (58). RAD54L+RAD54B depletion resulted in a significant increase in RAD51 in PCNA-negative (5 ± 5 versus 2 ± 2 RAD51 foci; *P* < 0.005) and PCNA-positive cells (13 ± 6 versus 9 ± 4; *P* < 0.05). This confirms our interpretation of the RPA staining data, showing definitively that RAD51 accumulation in translocase-depleted cells is not restricted to S phase. Similar results were obtained in S33 cells overexpressing RAD51 (data not shown). Metaphase and anaphase chromosomes were also decorated with RAD51 fibers, providing further evidence that these structures are not restricted to S-phase (as described below).

### RAD54L+B depleted MDAMB231 and PC-3 cells accumulate nondamage-associated RAD51 foci

We verified the results in MCF7 cells by determining the effect of RAD54L+RAD54B depletion in two additional cell lines: the prostate cancer cell line, PC-3 and the triple-negative breast cancer cell line, MDAMB231 (Supplementary Figure S5). In MDAMB231 cells, translocase depletion with either siRAD54L+RAD54B (22 ± 21 RAD51 foci/nucleus) or siRAD54L+RAD54B-2 (25 ± 19 RAD51 foci/nucleus) significantly increased RAD51 focus counts when compared to controls (7 ± 9 RAD51 foci/nucleus, *P* < 0.001). As was the case with MCF7 cells, depletion of translocases did not increase 53BP1 focus counts (8 ± 8 foci, siNS, 6 ± 4 foci siRAD54L+RAD54B-2, 7 ± 4 siRAD54L+RAD54B 53BP1 foci/nucleus). RAD54L+RAD54B depletion in PC-3 cells increased the number for RAD51 foci 2-fold from 6 ± 5 foci/nucleus in controls to 12 ± 7 foci/nucleus, but did not have an effect on 53BP1 focus counts (7 ± 5 versus 4 ± 5; *P* < 0.001; Supplementary Figure S5d–f). These results indicate that the ability of RAD54 to limit RAD51 focus accumulation can be seen in multiple tumor lines.

Previously, we demonstrated that PC-3 cells accumulated RAD51 on undamaged DNA when treated with RS-1, a small molecule that enhances the ability of RAD51 to bind both ss and dsDNA (36,59). Cells were further sensitized to RS-1 treatment by depletion of the RAD54 translocases (36). This led us to hypothesize that combining depletion of translocases with RS-1 treatment might further increase RS-1-induced RAD51 foci. To test this, we examined RS-1-induced RAD51 accumulation in cells depleted of RAD54L+RAD54B (Supplementary Figure S5d–f). Treatment of RAD54L+RAD54B depleted cells increased the average RAD51 focus counts from 26 ± 11 to 41 ± 18 foci/nucleus compared to RS-1 treatment alone (*P* < 0.001). Strikingly, 71% of RAD54L+RAD54B nuclei contained greater than 30 RAD51 foci/nucleus after RS-1 treatment compared to just 38% of nuclei with RS-1 treatment alone (*P* = 0.002). Thus, RAD54L+RAD54B depletion combined with RS-1 treatment increases RAD51 focus accumulation.

### Human RAD54 dissociates RAD51 from dsDNA in a purified system

Studies (60,61) have established that yeast Rad54 has the fundamental biochemical activity of disassembling yeast Rad51-dsDNA filaments. However, evidence for the human RAD54 disassembly of RAD51 filaments *in vitro* remained to be demonstrated. To this end, we used the modified electrophoretic mobility shift assay previously employed with the yeast system (60,61). In this assay (Figure 4a), Rad51 filaments are formed on radiolabeled dsDNA and then excess unlabeled ‘scavenger’ dsDNA is added to bind any Rad51 that disassembles from the original nucleoprotein filaments and thus prevent their reassembly on the original dsDNA. As shown in Figure 4b, yeast Rad51-dsDNA filaments enter the gel as a discrete species and are disassembled by Rad54 in a time-dependent manner to produce freely migrating dsDNA. Under the same magnesium-ATP conditions, human RAD51–dsDNA complexes do not enter the gel and remain in the well as aggregated species. It is not clear if these represent individual RAD51 filaments, complex filament networks or nonspecific DNA co-aggregates. RAD51-ssDNA filaments formed under similar conditions are unstable and are not productive for DNA strand exchange, while the addition of calcium ions inhibits RAD51 ATP hydrolysis and allows formation of extended, stable filaments (62). When calcium is included in the reactions (Figure 4b, c), RAD51-dsDNA filaments now enter the gel as a discrete species, and these filaments show very little turnover as evident by a lack of increase in the level of free substrate with time. The addition of RAD54 causes the nucleoprotein complexes to supershift into the wells. While the disassembly of RAD51-dsDNA filaments is inefficient at higher calcium concentrations (2 mM), titrating down the calcium concentration allows a progressive increase in the amount of free dsDNA that is liberated by RAD54 in a time-dependent manner (Figure 4c). This behavior is analogous to experiments with yeast proteins, where both the Rad54 and Rad51 ATPase activities are required for optimal disassembly of Rad51-dsDNA filaments (61). To test the possibility that RAD54 might also cause filament disassembly on ssDNA, we performed the assay under the low calcium (0.5 mM) condition and formed RAD51 filaments on a 705 nt ssDNA substrate (Figure 4d). After 2 h of incubation, all ssDNA remained bound by RAD51 and there was no significant difference in the migration of nucleoprotein species with or without RAD54. Taken together with the observation that ssDNA without secondary structure does not support ATP hydrolysis by RAD54 (18), these data demonstrate that RAD54 removes RAD51 specifically from dsDNA and not ssDNA.

### Translocase depletion does not alter RAD51 protein levels

Taken together, the results described above strongly suggest that an imbalance of RAD51 and RAD54L+RAD54B levels results in the accumulation of a dsDNA-bound form of RAD51. Given that more extensive RAD51 structures form in S33 cells upon RAD51 overexpression than in the other tumor lines in which RAD51 levels are not artificially elevated, the results suggest that induction of RAD51 overexpression in S33 yields a relatively high level of RAD51 expression. Indeed, analysis of RAD51 expression levels by western blotting showed that induction of RAD51 overexpression in S33 cells increases RAD51 to 4 μM while the level of expression of RAD51 was 9- to 13-fold lower in the other lines: 0.32 μM in MCF7, 0.35 μM in PC-3, 0.47 μM in MDAMB231 cells (Supplementary Figure S1a). Importantly, depletion of RAD54L+RAD54B did not result in an alteration in RAD51 levels in any of the cell lines, indicating that observed increases in RAD51 fibers and foci result from redistribution of the protein in the nucleus.

### The level of RAD51 expression observed in tumor lines is sufficient to promote substantial binding of RAD51 to dsDNA

RAD51 was previously shown to bind dsDNA directly by electrophoretic mobility shift assay (63). Similar results were obtained in a study of yeast Rad51 using a solution assay (1). We confirmed and extended this observation for human RAD51 using a solution DNA binding assay (59). We measured binding of RAD51 to a 162 bp dsDNA duplex substrate with hairpin DNA ends to prevent strand dissociation. We found that 0.32 μM RAD51 was sufficient to detect 64% of the maximal dsDNA binding (Supplementary Figure S6). Thus, the concentration of RAD51 in the human tumor cells examined here exceeds that required for direct binding to dsDNA *in vitro*.

### Induction of nondamage-associated RAD51 foci is associated with reduced cell growth

Next, we studied the impact of RAD51 overexpression and RAD54L+RAD54B depletion on cellular growth. RAD51 overexpression in S33 cells has previously been shown to decrease plating efficiency (6). To extend this observation, we monitored the ability of cells to perform DNA synthesis (i.e. to incorporate EdU) following changes in RAD51 and/or translocase levels. Cells pulsed with EdU for 45 min were subsequently quantified for EdU staining (Figure 5a). Both RAD51 overexpression and translocase depletion decreased the fraction of EdU-positive S33 cells about 1.5-fold (30 ± 4% and 28 ± 4%, respectively) relative to control cells (44 ± 4%). Furthermore, the combination of the two treatments resulted in a synergistic block to proliferation; the EdU-positive fraction was reduced 4.5-fold (10 ± 2%; *P* < 0.0005) compared to the control. In MCF7 cells, translocase depletion similarly resulted in a significant reduction in the EdU positivity (Figure 5a), with a striking 4.7-fold reduction at 72 h (10 ± 7% of translocase-depleted cells versus 47 ± 9% of control cells; *P* < 0.05). These results suggest that the accumulation of RAD51 complexes on undamaged chromosomes blocks growth.

### Replication defects associated with accumulation of nondamage-associated RAD51 complexes

The finding that reduction of translocase activity is associated with reduced proliferation raised the possibility that replication is blocked. To test this possibility, we employed a nascent DNA fiber technique to compare the efficiency of replication with reduced translocase levels to controls. Cells were pulsed labeled with IdU (red) for 20 min followed by a second 20-min pulse with CldU (green). DNA fiber spreads were prepared and fork progression examined by immunostaining. The majority of DNA fibers comprised adjacent IdU and CldU signals indicating continuous replication fork elongation during both pulses (Figure 5b). To determine if accumulation of RAD51 complexes is associated with an increase in fork stalling, we determined the percentage of forks containing IdU only (red) (64). In S33 cells, RAD51 overexpression resulted in a 2-fold increase in stalled replication forks compared to controls (11 ± 2% versus 5 ± 2%; *P* < 0.05). Depletion of RAD54L+RAD54B in cells overexpressing RAD51 further increased the percentage of stalled replication forks (19 ± 2%; *P* < 0.001). In MCF7 cells, RAD54L+RAD54B depletion resulted in a similar increase in stalled replication forks compared to controls (23 ± 2% versus 8 ± 2%). Together, these results indicate that accumulation of RAD51 foci and fibers is associated with replication blockage.

Next, we measured fork progression by measuring the CldU/IdU tract length ratio. A decrease in replication fork progression results in shorter CldU tracts compared to IdU (lower CldU/IdU ratio; Figure 5c; (38,65). RAD54L+RAD54B depletion had no effect on replication progression in cells with lower levels of RAD51 (CldU/IdU ratio: 0.8 in siRAD54L+B versus 0.76 in NS, respectively). Induction of RAD51 overexpression significantly reduced replication fork progression (CldU/IdU; 0.67; *P* < 0.01). Replication fork progression was further decreased by depletion of RAD54L+RAD54B (CldU/IdU: 0.6, *P* < 0.001). In MCF7 cells, RAD54L+RAD54B depletion also significantly decreased replication fork progression (CldU/IdU ratio: 0.47) compared to controls (CldU/IdU: 0.68, *P* < 0.001; Figure 5d). Together, these data suggest that accumulation of DNA bound RAD51 impairs replication fork progression by decreasing replication fork speed and/or increasing the frequency of replisome stalling.

### Mitotic defects associated with accumulation of nondamage-associated RAD51 complexes

In *Saccharomyces cerevisiae*, Rad51 accumulation on mitotic chromosomes has been shown to cause chromosome loss (31). To determine whether nondamage-associated RAD51 complexes similarly cause chromosome instability in human cells, we examined nuclear morphology in S33 and MCF7 cells. In S33 cells with lower RAD51, approximately 10 ± 4% of nuclei contained at least one micronucleus, but other abnormalities were not seen. By contrast, when RAD51 was overexpressed 80–86% of control and translocase-depleted cells displayed dramatic defects in nuclear morphology; filament-containing nuclei were multinucleate, contained micronuclei or were connected by DNA bridges (Figure 6a). We then examined the effects of RAD54L+RAD54B depletion in MCF7 cells. MCF7 cells depleted of RAD54L+RAD54B displayed a higher frequency of micronuclei (20 ± 2%) compared to controls (5 ± 1%), indicating that this cell line requires normal expression of RAD54L+RAD54B for genome stability (Figure 6a).

Multinucleate cells can arise as a result of severe defects in chromosomes condensation and segregation (66–68). Therefore, we sought evidence for defects during metaphase and anaphase in S33 and MCF7 cells containing nondamage-associated RAD51 complexes by immunostaining cells for the mitotic spindle component, α-tubulin. Chromosomes appeared undercondensed in 27 ± 6% of metaphase nuclei in S33 cells following RAD51 overexpression, compared to 0% of metaphase nuclei expressing lower RAD51 levels (Figure 6b,d). Combining RAD51 overexpression with translocase depletion further increased the percentage of mitotic figures with condensation defects 2-fold (52 ± 7%; *P* < 0.05). We verified this result by depleting cells with siRAD54L+RAD54B-2 siRNAs and found that translocase depletion increased the percentage of mitotic figures with condensation defects 2.3-fold (75 ± 33% siRAD54L+RAD54B-2; 33 ± 10% siNS; *P* < 0.05; Supplementary Figure S7a). Strikingly, RAD51 fibers were visible on 100% (N = 43) of metaphases with condensation defects. Translocase depletion did not result in detectable condensation defects in S33 cells that had not been induced to overexpress RAD51, nor was this the case in MCF7 cells. Together, these data indicate that extensive accumulation of RAD51 fibers on mitotic chromosomes blocks normal chromosome condensation during mitosis.

We then examined chromosome segregation during anaphase (Figure 6c). Mitotic cells that failed to separate into two distinct daughter nuclei were scored as containing segregation defects. They were further separated into two distinct classes: cells containing a single lagging chromosome or anaphase bridge (Class I), and those with multiple lagging chromosomes (Class II, Figure 6e). Overexpression of RAD51 resulted in an increase in the frequency of mitotic defects from 23 ± 6 to 59 ± 7 (*P* < 0.05). Translocase depletion combined with RAD51 overexpression resulted in a dramatic 4-fold increase in the frequency of Class II defects from 14 ± 9% to 55 ± 15% of anaphases (Figure 6e). A second set of siRNAs against RAD54L+RAD54B also promoted a similar increase in the frequency of Class II defects (Supplementary Figure S7b). These data indicate that RAD54L+RAD54B promote chromosome segregation in cells overexpressing RAD51. Increases in segregation defects were also observed without artificial overexpression of RAD51. In translocase depleted MCF7, 27 ± 6% of cells exhibited Class I segregation defects compared to 3.5 ± 2.4% of control cells (*P* < 0.005; Figure 6c,e). In MDAMB231 cells, 38 ± 10% of cells depleted for RAD54L+RAD54B exhibited Class I segregation defects compared to 17 ± 8% of controls and 4 ± 4% of nuclei exhibited Class II defects (Supplementary Figure S7c). Thus, conditions that promote accumulation of nondamage-associated RAD51 complexes, either by RAD51 overexpression and/or RAD54L+RAD54B depletion, generate genome instability *via* segregation errors.

## DISCUSSION

Here, we provide evidence that human RAD54 family translocases function to limit accumulation of DNA-bound RAD51 complexes in the absence of induced DNA damage. The data support a model in which RAD51 binds undamaged sites of dsDNA, rather than being recruited to sites of damaged DNA containing ssDNA tracts. This interpretation is consistent with the known dsDNA binding activity of RAD51 (1,69) and the concentration of RAD51 in the cell (as discussed further below). The interpretation is also supported by our demonstration of hRAD54's ability to dissociate RAD51 from dsDNA, which is in agreement with previous work on the orthologous yeast protein (60,61). The data presented argue that the RAD54 family translocases have an evolutionarily conserved function that prevents the accumulation of potentially toxic RAD51–dsDNA complexes (31,32). We propose that this function of RAD54 family translocases is important for genome stability because accumulation of RAD51 complexes on dsDNA is associated with inhibition of replication fork progression and interruption of proper chromosome segregation.

### RAD51 fibers may be helical nucleoprotein filaments

Previous work in S33 cells showed that RAD51 fibers form as a consequence of RAD51 overexpression. It was not clear, however, if these fibers were a DNA-bound form of the protein or not. We present three lines of evidence consistent with the notion that the fibers are bound to DNA: (i) they are sensitive to DNAse treatment, (ii) they co-localize with DNA-staining threads that are seen connecting nuclei following translocase depletion and (iii) their length and abundance increases when dsDNA-specific DNA translocases are depleted. We speculate that RAD51 fibers are the helical RAD51 nucleoprotein filaments observed in purified systems. Given that helical dsDNA-RAD51 nucleoprotein filaments contain 2.5 kb per micron, the assumption that fibers correspond to helical filaments implies that they involve tracts of DNA that are on the order of 5 kb in length. RAD51 and other members of the RecA family are well known to form such long filaments; indeed, such filaments have been extensively characterized biochemically (69–71). Although we favor the hypothesis that RAD51 fibers observed *in vivo* are composed of helical filaments, further studies are required to determine their molecular structure.

### RAD51 cellular concentration is sufficient to promote direct binding to dsDNA

We estimated the cellular concentration of RAD51 in human tumor cells and found that the concentration in MCF7 is about 0.32 μM, a concentration at which purified RAD51 displays significant binding to dsDNA. Given the complexity of chromatin, it is difficult to estimate the concentration of nuclear dsDNA segments available to bind RAD51 directly. However, the concentration of genomic DNA in a mammalian nucleus can be estimated to be about 10 mM bp (based on a diploid genome size of 6.5 Gbp and an average nuclear volume of 1.0 x 10^−6^ μl). Thus, the concentration of RAD51 in the nucleus is sufficient to promote direct dsDNA binding even if less than 1/10,000 of the dsDNA is accessible. We view these data as supporting the proposal that the RAD51 foci observed in translocase depletion experiments arise as a consequence of direct binding of RAD51 to dsDNA.

### The effects of RAD51-RAD54 imbalance

We examined the effects of translocase depletion in three different contexts that we believe differ in the degree of imbalance between RAD51 dsDNA association and tranlocase-mediated dissociation. In S33 cells with low levels of RAD51, RAD54L+RAD54B depletion leads to a minor imbalance resulting in a slight, but significant accumulation of RAD51 foci specifically in S-phase cells. In S33 cells, artificial induction of very high levels of RAD51 protein leads to a much greater imbalance resulting in the accumulation of long RAD51 fibers (6). These abnormal structures become longer and somewhat more numerous when translocase activity is depleted. In human tumor cells such as MCF7, RAD51 forms higher levels of spontaneous foci that most often appear as simple resolution-limited staining foci (14). If such foci represent canonical RAD51 helical filaments, their contour lengths must be less than 400 bp, given the resolution of the imaging method. Experimental reduction of translocase expression in MCF7, PC-3 and MDAMB231 cells results in a moderate imbalance between RAD51 and RAD54 activity, increasing the number of RAD51 foci and resulting in the accumulation of a small number of fibers.

Differing severity of chromosomal abnormalities caused by RAD51 overexpression or RAD54L+RAD54B depletion is consistent with being caused by differing degrees of imbalance of RAD51 and RAD54L+RAD54B activity. Depletion of translocases in MCF7 cells, which results in a modest accumulation of RAD51 structures, is associated with relatively modest anaphase abnormalities that typically appear to involve a single lagging chromosome (72). Induction of very high levels of chromosome-associated RAD51 complexes is associated with more severe chromosome abnormalities. Indeed, severe defects in chromosome condensation and segregation has been shown previously to lead to severe abnormalities in nuclear morphology (66–68,73). In the case of cells induced to express artificially high levels of RAD51, no additional increase in the severity of associated defects was detected by translocase depletion, a result we interpret to suggest that artificial overexpression of RAD51 in S33 cells results in the maximum defect observable in our phenotypic assays.

In contrast to the correspondence of the severity of segregation defects to the level of RAD51-RAD54 imbalance in overexpressing S33 cells and MCF7 cells, the replication assay we employed showed similar defects caused by translocase depletion in the two types of cells. However, this result is likely to be misleading due to the fact that the DNA combing assay is only capable of detecting defects at sites still engaged in replication at the time of DNA labeling; if most forks are blocked at the time of labeling in S33, the assay would not necessarily have detected a relatively severe replication defect in overexpressing S33 cells. Supporting this interpretation is the observation that high levels of RAD51 expression in S33 cells result in the redistribution of RPA from the relatively diffuse pattern associated with normal S phase to the punctate pattern that is characteristic of a severe replication blockage (48,49). No such RPA redistribution was observed in translocase-depleted MCF7 cells, suggesting that the replication blockage is not as severe in that situation. Alternatively or in addition to direct effects on replication fork stalling, accumulation of RAD51 on undamaged DNA may result in a significant reduction in the free pool of RAD51 available for repair of stalled/collapsed replication forks. Previous studies have indicated RAD51 protects replication forks and mediates replication fork restart in response to hydroxyurea (64,74). Thus, the replication defects observed in cells containing nondamage-associated RAD51 complexes may arise due to sequestration of RAD51, and/or RAD51-associated repair factors, away from stalled replication forks.

### The nature of RAD51-mediated segregation defects

At least some of the segregation defects observed in cells containing nondamage-associated RAD51 complexes may arise due to cells entering mitosis with incompletely replicated DNA. Previous studies have demonstrated that reduction of replication fork speed results in segregation errors (75,76). Alternatively or in addition, the presence of RAD51 complexes bound to DNA during metaphase and anaphase may block topoisomerase II activity resulting in accumulation of catenated DNA. Indeed, the phenotypes observed in S33 and MCF7 cells are reminiscent of topoisomerase II depleted cells, which also display mitotic condensation defects, increased micronucleus formation, increased multinucleated cells, chromosome segregation defects and PICH-positive DNA bridges (73).

### Implications for cancer

RAD51 overexpression has been observed in a wide variety of cancer types (8–14,77) and spontaneous RAD51 foci have been reported in various cancer cell lines (14). Our data support a model in which inhibition of RAD54L+RAD54B destabilizes the genome under conditions that promote the accumulation of toxic DNA-bound RAD51 complexes. These findings indicate that imbalance of RAD51-dsDNA binding and translocase-mediated RAD51 dissociation is genome destabilizing. Thus, in addition to its genome stabilizing DNA repair activity, RAD51 may also promote tumor progression in tumors where RAD54 activity is limiting. Because RAD51 overexpression and spontaneous RAD51 foci are common features of malignant cells, our findings suggest a possible role for nondamage-associated RAD51 complexes in malignant progression. A second oncologic implication is that the balance of RAD51-RAD54 activities may represent a drug target for cancer treatment because tumor cells expressing high levels of RAD51 may prove to be differentially sensitive to compounds such as streptonigrin that inhibit RAD54 (78). Proof in principle for this targeting strategy is provided by RS-1, which enhances toxic RAD51 activity (36).

## SUPPLEMENTARY DATA

Supplementary Data are available at NAR Online.

SUPPLEMENTARY DATA
