# Supplementary material for: RAD54 family translocases counter genotoxic effects of RAD51 in human tumor cells
Source: Nucleic Acids Res. 2015 Mar 12;43(6):3180–96. doi: 10.1093/nar/gkv175 (PMC4381078; doi:10.1093/nar/gkv175)
Supplement: SUPPLEMENTARY DATA [file supp_43_6_3180__index.html]

RAD54 family translocases counter genotoxic effects of RAD51 in human tumor cells — RAD54 family translocases counter genotoxic effects of RAD51 in human tumor cells — SUPPLEMENTARY DATA 

# RAD54 family translocases counter genotoxic effects of RAD51 in human tumor cells

## SUPPLEMENTARY DATA

**Files in this Data Supplement:**

- SUPPLEMENTARY DATA
