# Supplementary material for: RAD54 family translocases counter genotoxic effects of RAD51 in human tumor cells
Source: Nucleic Acids Res. 2015 Mar 12;43(6):3180–96. doi: 10.1093/nar/gkv175 (PMC4381078; doi:10.1093/nar/gkv175)
Supplement: SUPPLEMENTARY DATA [file supp_gkv175_nar-01990-h-2014-File003.pdf]

Mason et al.

RAD54 family translocases counter genotoxic effects of RAD51 in human tumor cells

Supplemental Material and Methods

DNA binding assay. Human RAD51 protein was purified as previously described (1). The fluorescence polarization assay to measure protein binding to DNA was performed as in (1) with modifications: Binding reactions were carried out at 37° C for 40 minutes in a buffer containing 20 mM HEPES pH 7.5, 2 mM ATP, 10 mM $MgCl_2$, 0.1 mM tris (2-carboxyethyl)phosphine (TCEP), 0.25 µM BSA, 30 mM NaCl, 2% glycerol. DNA substrates containing a single fluorescent label were supplied at 0.5 µM nucleotide or base pair concentration. The 84-nt ssDNA substrate is the RG1(+) substrate used previously by Gupta et al (2) with the 5' fluorescein tag substituted with an Alexa 488 fluorophore. The 162-bp dsDNA substrate is DHD162, described previously (3). Fluorescence polarization was read using a Tecan infinite 200Pro plate reader equipped with 485 +/- 20 nm excitation and 535 +/- 25 nm emission filters.

siRNA depletion. A cocktail consisting of three independent siRNAs targeting RAD54L (A-C) was purchased from Santa Cruz. Six independent siRNAs against RAD54B (1-6) and three independent siRNAs against RAD54L (1-3) were purchased from Invitrogen. The All-stars non-silencing (NS) siRNA was used as a control (Qiagen). RAD54L (A-C) siRNA complexes were transfected to a final concentration of 25 nM and RAD54B (1-3) was transfected to a final concentration of 50 nM using Lipofectamine RNAiMax (Invitrogen) as per manufacturer's instructions. For siRAD54L+B-2 transfections, RAD54L (1-3) siRNA were transfected to a final concentration of 25 nM and RAD54B (4-6) were transfected to a final concentration of 75 nM.

The following siRNA sequences were used in this study:

siRAD54L+B:

RAD54B-1 5'CCTCATTAGCCTTTCTTGTGAGAAA

RAD54B-2 5' GCTAGGAAGTGAAAGGATCAAGATA

RAD54B-3 5' GACATTGGAAGAGGCATTGGTTATA

RAD54L-A 5' GATCTGCTTGAGTATTTCA

RAD54L-B 5' CCGTAGCAGTGACAAAGTA

RAD54L-C 5' GAACCCAGCCAATGATGAA

siRAD54L+B-2:

RAD54B-4 5' GCTTACGGCATGATCTACCTGAATA

RAD54B-5 5' TGGGTATCATAGCAGTTAACTATA

RAD54B-6 5' CAGCACAGGGCCTACGCACCCTTTA

RAD54L-1 5' GGAAACCTTTGAGTCAGCTAACCAA

RAD54L-2 5' TTGGTTAGCTGACTCAAAGGTTTCC

RAD54L-3 5' GCCAAGGTTGTAGAACGCTTCAATA

Competitive PCR. We employed competitive PCR to determine the amount of depletion of mRNA. RNA was harvested from cells using a High Pure RNA Isolation Kit (Roche). 0.5 μg of total RNA was reverse-transcribed using MLV-RT (Qiagen) with a dT (20) primer at 42°C for 1 hour. To quantitate mRNA levels, competitive PCR was performed as previously described (4). Briefly, competitor fragments for RAD54L and RAD54B were generated by inserting a 20 bp sequence (5' GTCGACGGATCCCTGCAGGT) into the product amplified by gene-specific PCR primers. The relative amount of RNA was determined by co-amplification of cDNA with increasing amounts of competitor fragments. Products were run on 2 % agarose gels in 1X TBE and band intensity was measured using ImageJ. The ratio of band intensity of Competitor (C) to target cDNA (T) was

determined and plotted. The amount of cDNA amplified in each reaction was determined by identifying the amount of competitor that resulted in a C/T ratio of 1. For all RT reactions, GAPDH was used as a loading control.

Determination of the concentration of RAD51 in the nucleus. MCF7 cells were harvested, counted, and lysed as above. Whole cells extract were loaded on an SDS-PAGE gel along with increasing amounts of human RAD51 protein. Protein was transferred to PVDF and probed with RAD51 antibody. Band intensity was quantitated using ImageJ. To calculate the concentration of RAD51 in tumor cells, the mass of RAD51 protein per lane was determined using a standard curve generated with purified RAD51 protein. The mass of RAD51/cells was then determined by dividing the mass per lane by the number of cell equivalents loaded. RAD51 concentration was then calculated using a nuclear volume of $1x10^{-12}$. This volume was calculated based on measurement of the average nuclear diameter (12.4 μm). We also assumed that 70% of total RAD51 is localized in the nucleus (5).

Antibodies. RAD51 antibody was a kind gift from A. Shinohara (1:1500). RPA antibodies (Na18 for human, Ab-3 for mouse) were from CalBioChem (1:2000). α-Tubulin (Ab-2) was from Fitzgerald (1:500). RAD54B was a kind gift from P. Sung (6) (1:1000). RAD54L (4E3/1, 1:500) and Rat anti-BrdU (BU1/75, 1:200) were from Abcam. PCNA (IG7, 1:500) and anti-ERCC6L/PICH (DO1P, 1:100) were from Abnova. 53BP1 (BP13) and γH2AX Ser319 (JBW301) were from Upstate. Lamin B (M-20) was from Santa Cruz (1:500) and mouse anti-BrdU (B44) was from Beckton Dickinson (1:25).

Proteins. Purification of yeast Rad51(7), yeast Rad54(8), human RAD54 (9), human RAD51(10) were performed as described.

Assay for RAD51 filament disassembly by RAD54. *Reaction conditions*: Reactions were carried out in a buffer containing 35 mM triethanoamine pH 7.5, 4 mM $MgCl_2$, 2 mM ATP, 1 mM DTT, 0.1 mg/ml BSA, 25 mM disodium phophocreatine, 0.1 mg/ml phosphocreatine kinase, and the indicated concentrations of $CaCl_2$ (all concentrations listed are after full dilution). In a volume of 18 μl, 6 μM bp of dsDNA or 6 μM nt of ssDNA were incubated with 1.5 μM RAD51 at 23 °C for 20 minutes. Then, 100 nM RAD54 was added (1 μl of 2 μM)

and the incubation continued for two minutes. At this time, 30 % of the reaction (5.7 µl) was removed and fixed with 0.25 % glutaraldehyde and immediately afterward 300 µM bp of *Xmn*1-linearized pUC19 DNA was added (0.7 µl of 600 µM) to the remainder of the reaction and incubation continued. At one and two hours past addition of pUC19 scavenger, 30 % of the reaction (6 µl) was removed and fixed with glutaraldehyde. About half an hour after fixation of the last time point, DNA loading dye was added to the samples, which were then loaded on a 1 % agarose TAE gel and electrophoresed for 100 minutes at 4 V/cm. Gels were then vacuum-dried on DE-81 (Whatman) paper and exposed to a storage phosphor screen overnight and imaged on a Storm 860 scanner. Reactions were quantitated using ImageQuant software by normalizing the freely migrating DNA species (non-shifted mobility) to an equivalent mass of ^{32}P DNA electrophoresed without protein as equal to 100 % free substrate. *DNA Substrates:* Linear dsDNA substrate (~3 kb) was generated by PCR amplification of pBluescript plasmid DNA, followed by ethanol precipitation. The DNA in the pellet was dissolved and passed through a PCR Kleen (BioRad) spin column to remove primers and excess dNTPs. The resulting DNA (about 10 µg) was 5' end labeled with 30 U of T4 polynucleotide kinase and 50 µCi $^{\gamma 32}$P-ATP in a volume of 200 µl. After 1 hour incubation at 37 °C, the reactions were purified with a PCR purification kit (Bioneer) and quantified with a Nanodrop spectrophotometer. 705 nt ssDNA was produced from phagemid circular ssDNA as described (11). This DNA was 5' end labeled and re-purified as described above for the dsDNA substrate. *Xmn*I-linearized pUC19 DNA 'scavenger DNA' was produced by plasmid digestion followed by two rounds of phenol/chloroform extraction, chloroform extraction and ethanol precipitation.

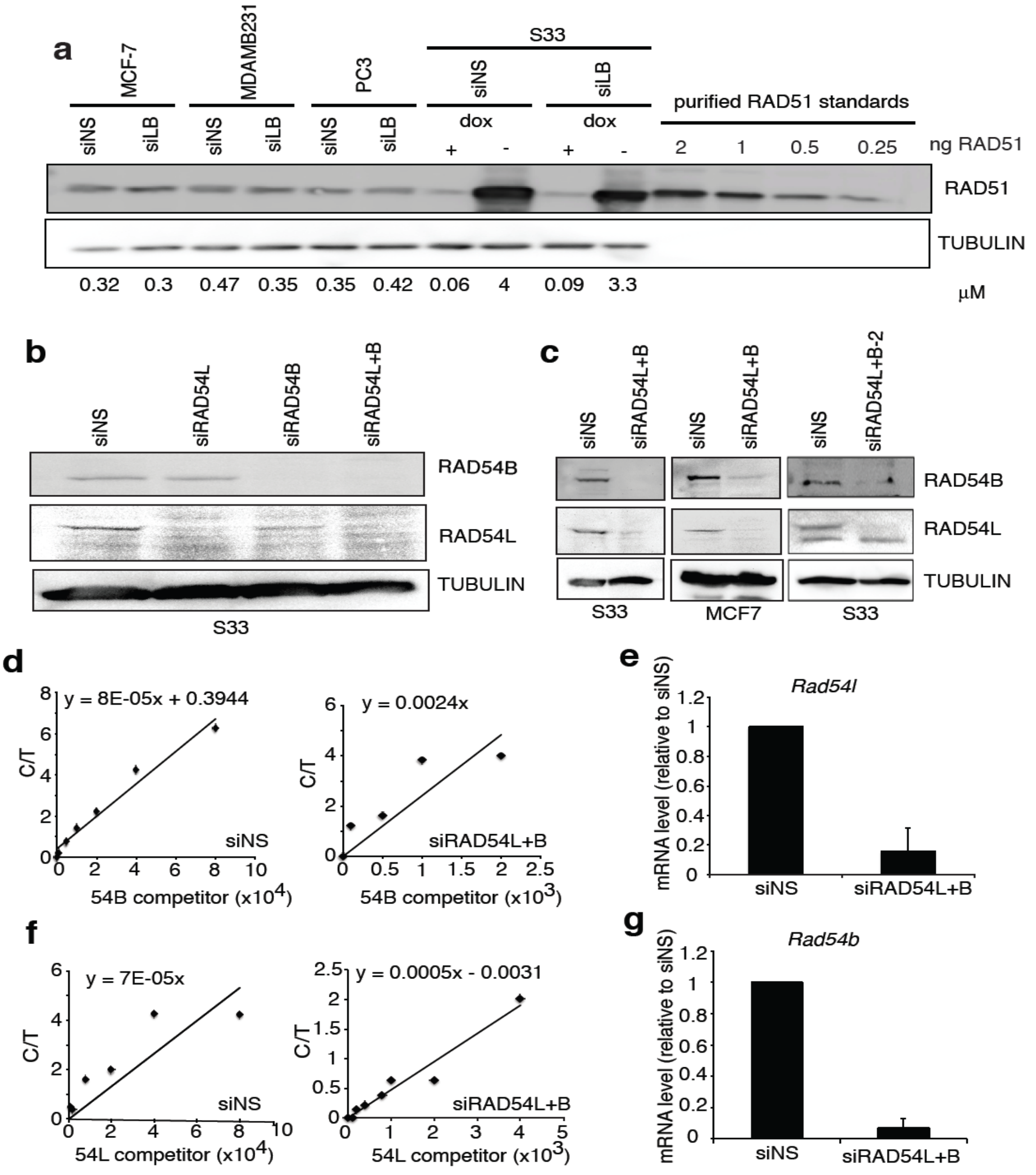

Supplemental Figure 1. Analysis of expression levels. (a) The concentration of RAD51 in tumor cells was estimated by quantitative western using decreasing amounts of purified human RAD51 protein as a standard curve. Whole cells extracts were loaded onto the western blot along with the indicated amount of purified human RAD51. The estimated cellular concentration of RAD51 is noted below the blot. α-Tubulin was used as a loading control. **(b,c)** Western blots depicting RAD54L and RAD54B protein levels after transfection with the indicated siRNAs. **(d)** Competitive PCR to determine mRNA levels after indicated siRNA transfections. PCR was carried out with increasing concentration of competitor (see Supplemental Material and Methods for details). Representative graph plotting the ratio of Rad54l band intensities between competitor (C) and target cDNA (T) in cells transfected with NS or RAD54L+B siRNAs. The amount of competitor used in the PCR reaction is depicted on the x-axis **(e)** Graph depicting the level of Rad54l mRNA after transfection with RAD54L+B siRNAs compared to the NS control. Graph depicts average of two independent reactions. **(f)** Representative graphs measuring *Rad54b* levels as described in c **(g)** Graph depicting the level of Rad54b mRNA after transfection with RAD54L+B siRNAs compared to the NS control. Graph depicts average of two independent reactions. Error bars are standard error.

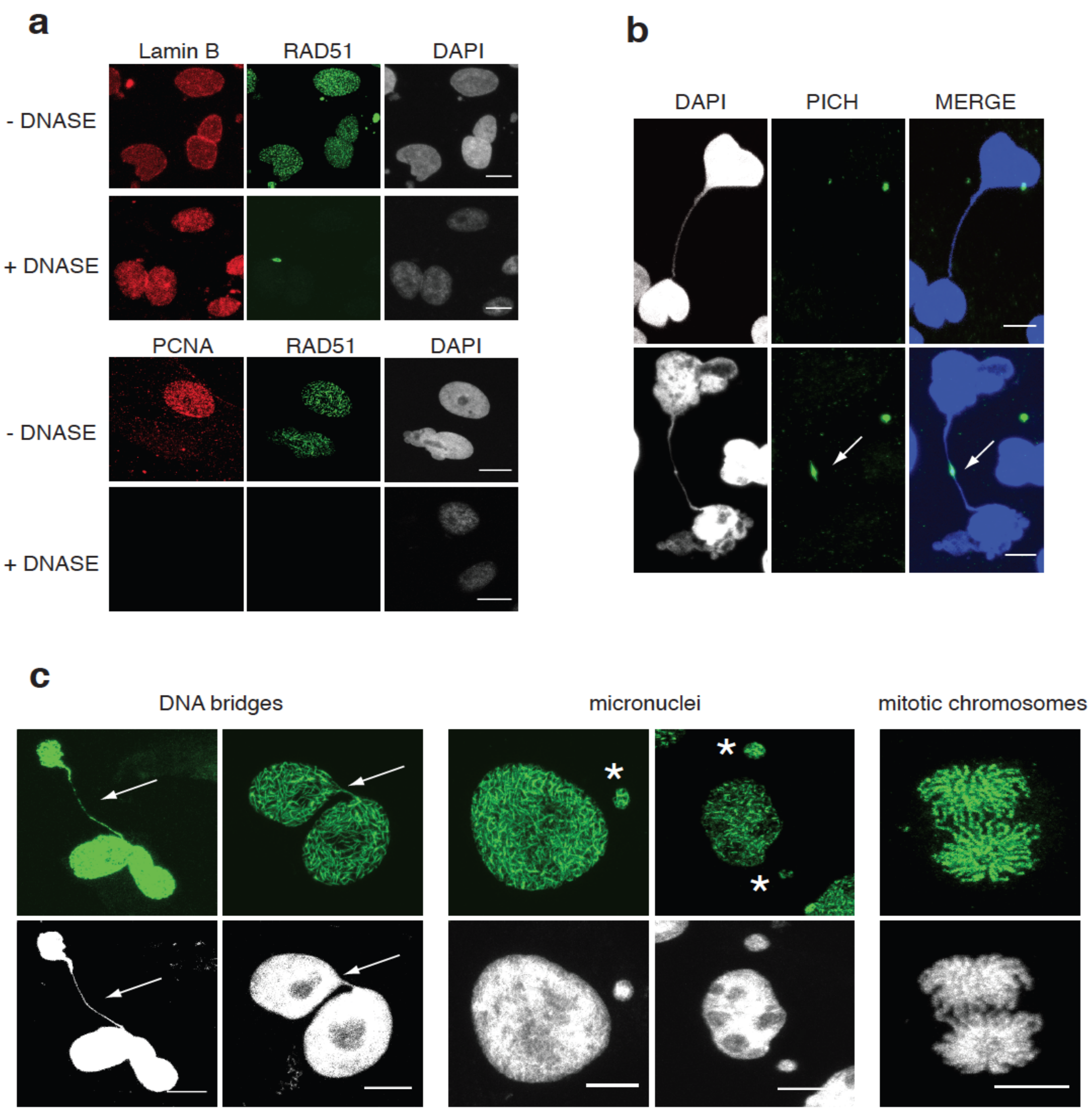

Supplemental Figure 2. RAD51 fiber analysis in S33 cells overexpressing RAD51. (a) Cells treated with DNaseI were stained for RAD51 (green) and either Lamin B (top panels) or PCNA (bottom panels). **(b)** PICH localization on DNA bridges in S33 cells overexpressing RAD51. Cells were stained with PICH (green). Top Panels: PICH negative DNA bridge. Bottom panel: PICH-positive DNA bridge. **(c)** Examples of RAD51 bound to DNA bridges (arrows), micronuclei (star) and mitotic chromosomes (far right image) in S33 cells overexpressing RAD51. Please note the examples depicted are from cells after RAD54L+B depletion. Brightness and contrast were adjusted to make the different structures readily visible. Scale bars: 10 μm.

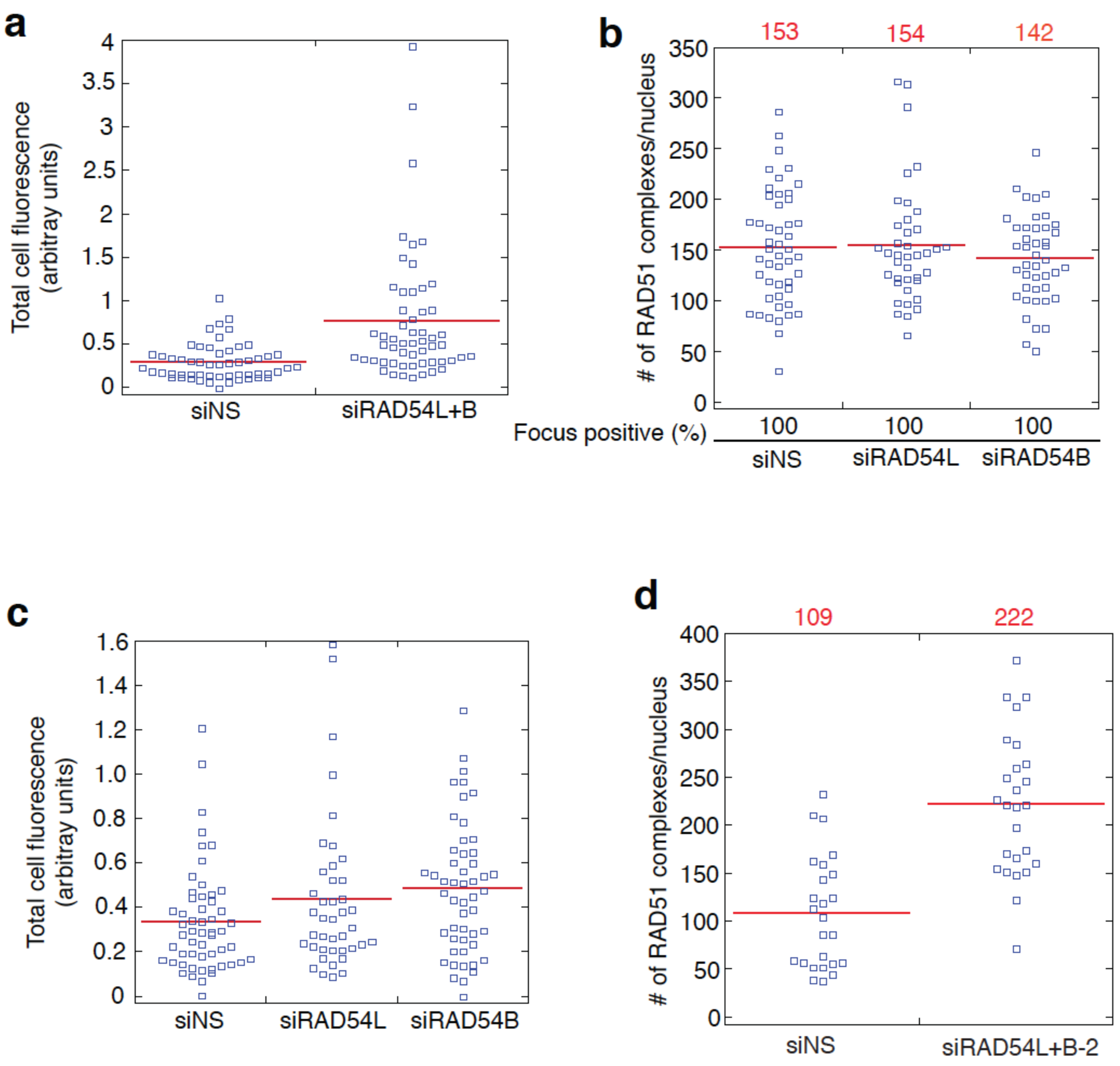

Supplemental Figure 3. RAD51 fiber formation in translocase depleted cells. (a) Dot plot depicting total fluorescence intensity of RAD51 staining in S33 HT1080 cells in RAD51 overexpressing cells transfected with siNS or RAD54L+B. Horizontal line represents mean. **(b)** Dot plot represents quantification of RAD51 complexes in S33 cells cultured in the absence of dox after depletion of either RAD54L or RAD54B alone. Red horizontal line represents the mean. Mean focus counts are reported above the graph in red letters. **(c)** Dot plot depicting total fluorescence intensity of RAD51 staining in siRAD54L and siRAD54B depleted cells as in (a). Graphs represent quantification of 50 random nuclei from two independent experiments. **(d)** Dot plot depicting RAD51 fiber counts in S33 cells after depletion of RAD54L+B with RAD54L+B-2 as done in (b).

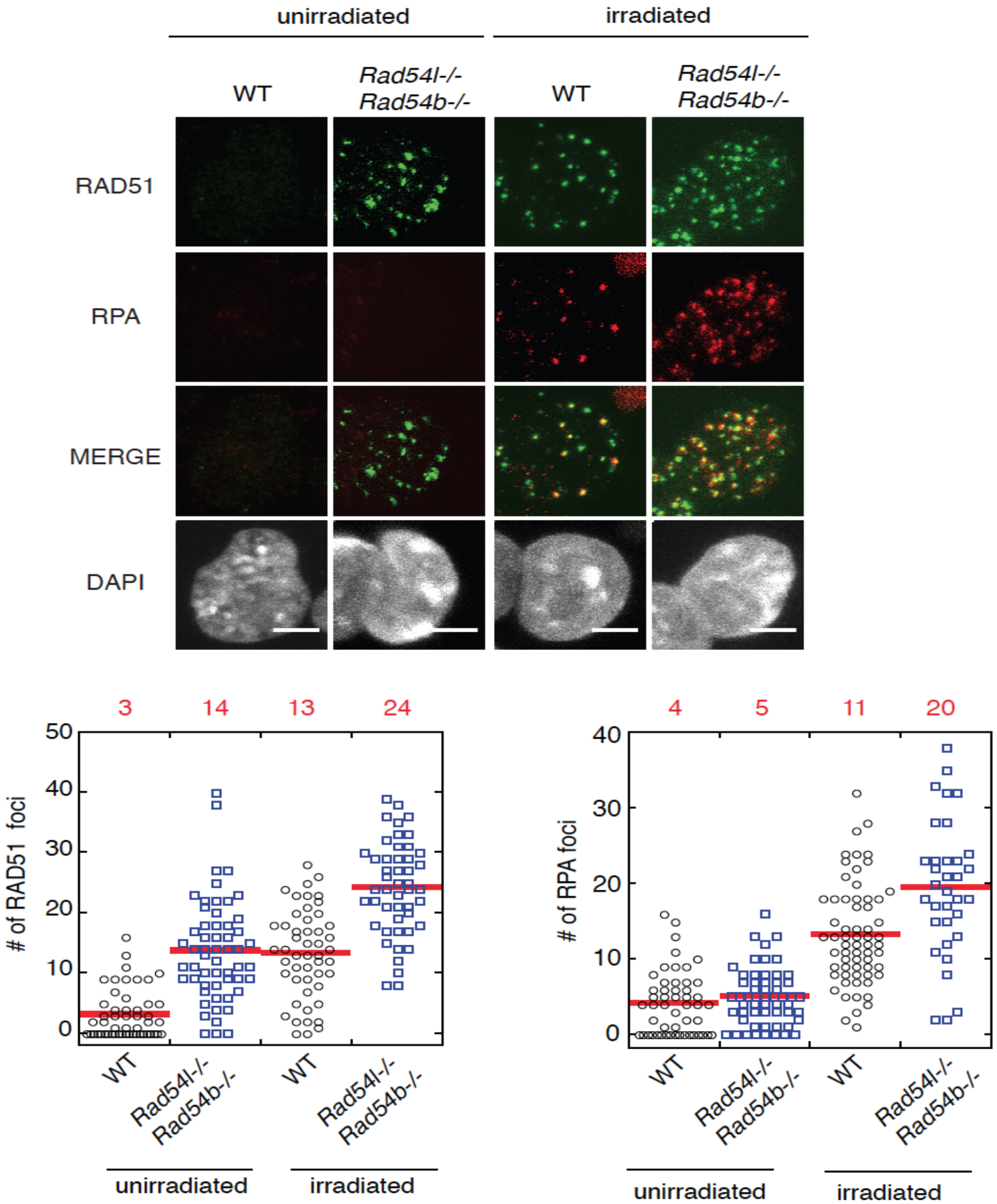

Supplemental Figure 4. RAD51 accumulates in *$Rad54l^{-/-}Rad54b^{-/-}$* mouse embryonic stem cells. Representative images depicting RAD51 and RPA localization in WT and RAD54 translocase-deficient ES cells. Red lines and numbers represent mean focus counts. Graphs represent quantitation of 50 random nuclei from two independent experiments. In unirradiated cells, *Rad54l-/-Rad54b-/-* ES cells contained on average 13±6 RAD51 foci/nucleus compared to WT cells (3±6 foci/nucleus). RPA focus counts in the mutant cell lines were not significantly different from those in WT ES cells (5±3 *Rad54l-/-Rad54b-/-* vs 4±5 WT). Irradiation significantly increased RPA focus counts in both *Rad54l-/-RAd54b-/-* and WT (20±8 and 11±5, respectively) and also elevated RAD51 focus counts (24±8 and 13±7, respectively). Scale bar: 10 μm.

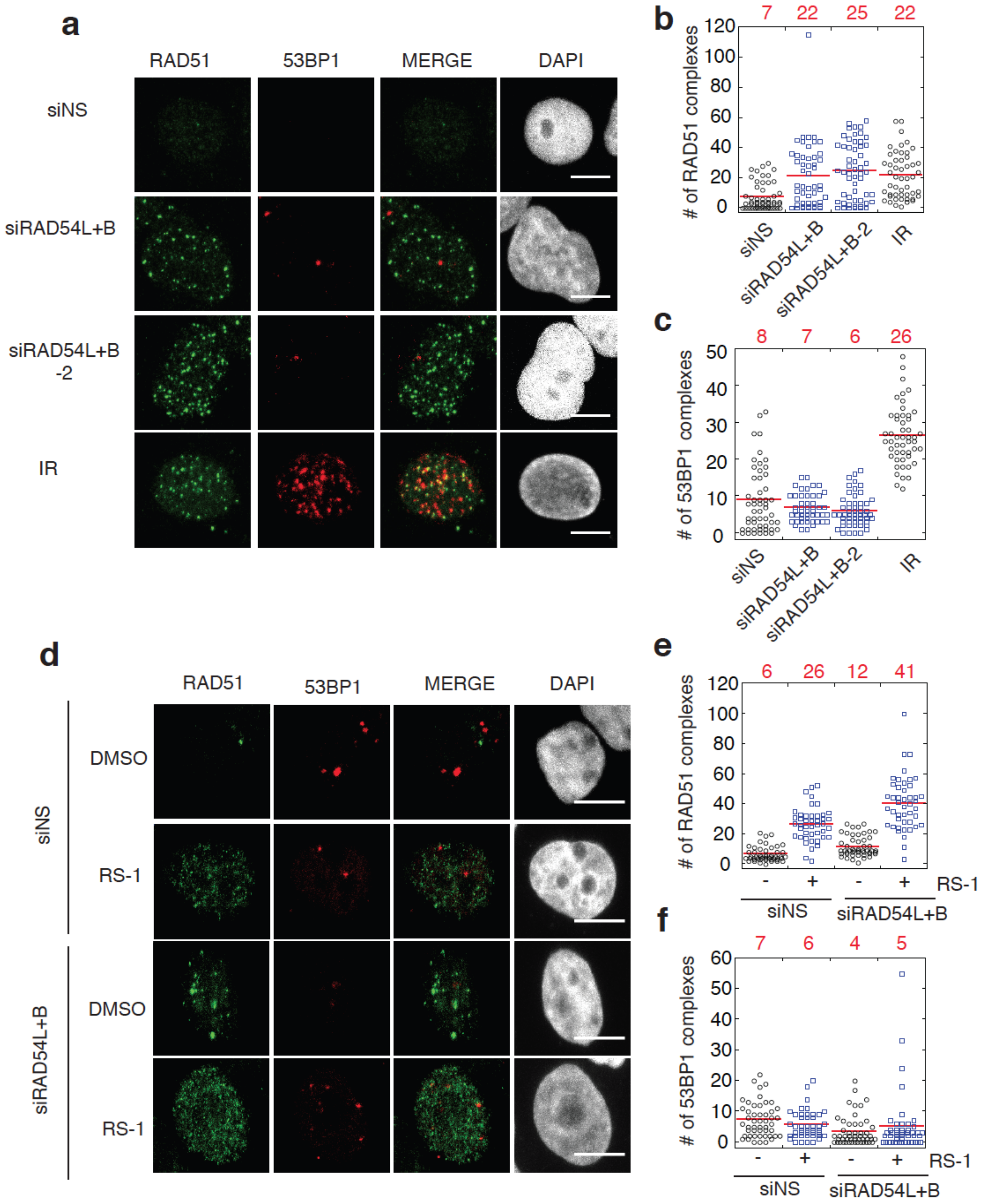

Supplemental Figure 5. RAD51 foci accumulate in translocase depleted MDAMB231 and PC3 cells. (a) Representative images of RAD51 (green) and 53BP1 localization (red) in MDAMB231 after the indicated treatments. **(b-c)** Dot plot depicting the number of RAD51 foci (b) or 53BP1 foci (c) after the indicated treatments. **(d)** RAD51 localization in PC-3 cells after the indicated treatments. Cells were treated with 60 uM RS-1 prior to fixation. Representative images depicting RAD51 (green) and 53BP1 are shown. **(e-f)** Dot plot depicting the number of RAD51 foci (e) or 53BP1 foci (f) after the indicated treatments. Red lines and numbers represent the Mean. Scale bar: 10 μm.

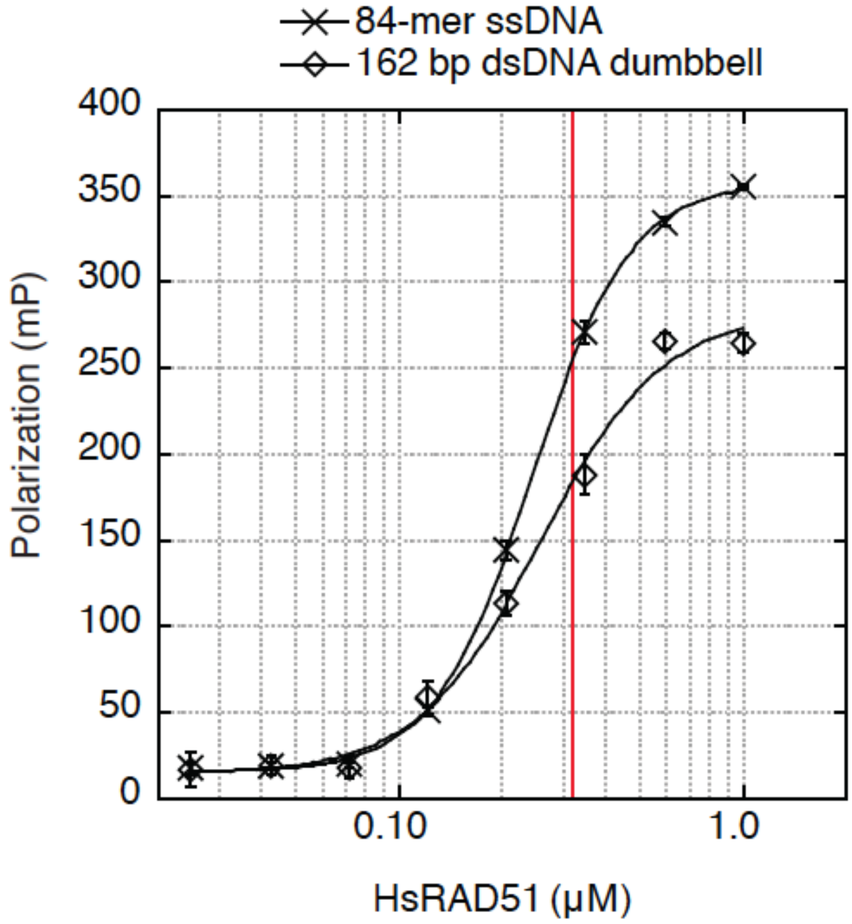

Supplemental Figure 6. DNA binding activity of purified human RAD51. Fluorescence polarization assay to detect hRAD51 binding to an 84-nt ssDNA or 162 bp dsDNA. hRAD51 was titrated against 0.5 uM nucleotide or base pair concentration of ssDNA or dsDNA. Protein binding to the fluorescently-labeled DNA results in increased fluorescence polarization. Red line denotes 0.32 uM RAD51 concentration. Error bars are standard error for four replicates.

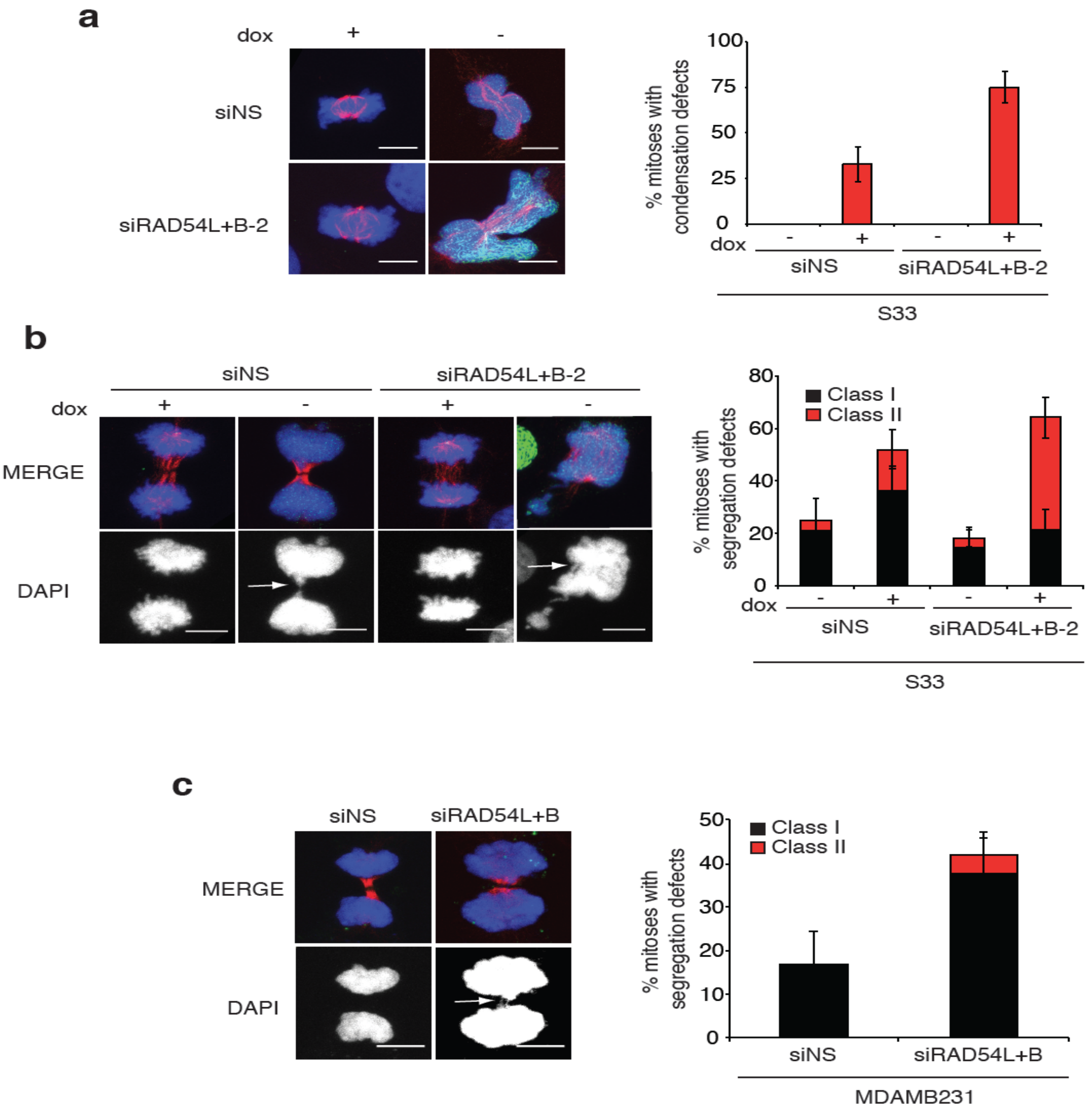

Supplemental Figure 7. Mitotic defects in S33 cells and MDAMB231 after translocase depletion. **(a)** Condensation defects in S33 cells overexpressing RAD51.Representative images depicting undercondensed chromosomes after depletion of translocases with siRAD54L+B-2. Graph represents the proportion of nuclei with condensation defects. **(b)** Representative images in S33 cells after the indicated treatments. Arrows indicate midzone regions with evidence for one or more lagging chromosome. Graph depicts the proportion of nuclei with Class I or Class II segregation defects (see text for details). **(c)** Segregation defects in MDAMB231 cells after depletion of RAD54L+B. Representative mitoses are depicted. Graph represents proportion of nuclei with Class I or Class II defects. Red: a-Tubulin, Green: RAD51, Blue: DAPI. Scale Bars: 10 µm. Error bars are standard error.

Supplemental References

1. Jayathilaka,K., Sheridan,S.D., Bold,T.D., Bochenska,K., Logan,H.L., Weichselbaum,R.R., Bishop,D.K. and Connell,P.P. (2008) A chemical compound that stimulates the human homologous recombination protein RAD51. *PNAS*, **105**, 15848–15853.

2. Gupta,R.C., Golub,E.I., Wold,M.S. and Radding,C.M. (1998) Polarity of DNA strand exchange promoted by recombination proteins of the RecA family. *Proc. Natl. Acad. Sci. U.S.A.*, **95**, 9843–9848.

3. Budke,B., Chan,Y.-L., Bishop,D.K. and Connell,P.P. (2013) Real-time solution measurement of RAD51- and RecA-mediated strand assimilation without background annealing. *Nucleic Acids Res.*, **41**, e130.

4. Zentilin,L. and Giacca,M. (2007) Competitive PCR for precise nucleic acid quantification. *Nat Protoc*, **2**, 2092–2104.

5. Essers,J., Hendriks,R.W., Wesoly,J. and Beerens,C. (2002) Analysis of mouse Rad54 expression and its implications for homologous recombination. *DNA Repair*.

6. Wesoly,J., Agarwal,S., Sigurdsson,S., Bussen,W., Van Komen,S., Qin,J., van Steeg,H., van Benthem,J., Wassenaar,E., Baarends,W.M., et al. (2006) Differential contributions of mammalian Rad54 paralogs to recombination, DNA damage repair, and meiosis. *Mol. Cell. Biol.*, **26**, 976–989.

7. van Komen,S., Macris,M., Sehorn,M.G. and Sung,P. (2006) Purification and assays of Saccharomyces cerevisiae homologous recombination proteins. *Meth. Enzymol.*, **408**, 445–463.

8. Kiianitsa,K., Solinger,J.A. and Heyer,W.D. (2002) Rad54 protein exerts diverse modes of ATPase activity on duplex DNA partially and fully covered with Rad51 protein. *J. Biol. Chem.*, **277**, 46205–46215.

9. Mazina,O.M. and Mazin,A.V. (2004) Human Rad54 protein stimulates DNA strand exchange activity of hRad51 protein in the presence of Ca2. *J. Biol. Chem.*, **279**, 52042–52051.

10. Hilario,J., Amitani,I., Baskin,R.J. and Kowalczykowski,S.C. (2009) Direct imaging of human Rad51 nucleoprotein dynamics on individual DNA molecules. *Proc. Natl. Acad. Sci. U.S.A.*, **106**, 361–368.

11. Wright,W.D. and Heyer,W.-D. (2014) Rad54 functions as a heteroduplex DNA pump modulated by its DNA substrates and Rad51 during D loop formation. *Mol. Cell*, **53**, 420–432.
